# Assessing the phylogenetic relationship among varieties of *Toona ciliata* (Meliaceae) in sympatry with chloroplast genomes

**DOI:** 10.1002/ece3.10828

**Published:** 2023-12-13

**Authors:** Yu Xiao, Xi Wang, Zi‐Han He, Yan‐Wen Lv, Chun‐Hua Zhang, Xin‐Sheng Hu

**Affiliations:** ^1^ College of Forestry and Landscape Architecture South China Agricultural University Guangzhou China; ^2^ Guangdong Key Laboratory for Innovative Development and Utilization of Forest Plant Germplasm Guangzhou China; ^3^ Institute of Highland Forest Science, Chinese Academy of Forestry Kunming China

**Keywords:** chloroplast genome, genetic conservation, purifying selection, sympatric speciation, *Toona ciliata*

## Abstract

*Toona ciliata* is an endangered species due to over‐cutting and low natural regeneration in China. Its genetic conservation is of an increasing concern. However, several varieties are recognized according to the leaf and flower traits, which complicates genetic conservation of *T. ciliata*. Here, we sequenced the whole chloroplast genome sequences of three samples for each of four varieties (*T. ciliata* var. *ciliata*, *T. ciliata* var. *yunnanensis*, *T. ciliata* var. *pubescens*, and *T. ciliata* var. *henryi*) in sympatry and assessed their phylogenetic relationship at a fine spatial scale. The four varieties had genome sizes ranged from 159,546 to 159,617 bp and had small variations in genome structure. Phylogenomic analysis indicated that the four varieties were genetically well‐mixed in branch groups. Genetic diversity from the whole chloroplast genome sequences of 12 samples was low among varieties (average *π* = 0.0003). Besides, we investigated genetic variation of 58 samples of the four varieties in sympatry using two markers (*psa*A and *trn*L‐*trn*F) and showed that genetic differentiation was generally insignificant among varieties (*Ф*_st_ = 0%–5%). Purifying selection occurred in all protein‐coding genes except for the *ycf*2 gene that was under weak positive selection. Most amino acid sites in all protein‐coding genes were under purifying selection except for a few sites that were under positive selection. The chloroplast genome‐based phylogeny did not support the morphology‐based classification. The overall results implicated that a conservation strategy based on the *T. ciliata* complex rather than on intraspecific taxon was more appropriate.

## Graphical abstract

This research used chloroplast genome sequences to assess the phylogenetic relationship of varieties of *Toona ciliata*, an endangered species in China. The results provided evidence of their close genetic relationship in sympatry and explored the potential evolutionary mechanism for a conflict between organelle‐ and morphology‐based phylogenies.

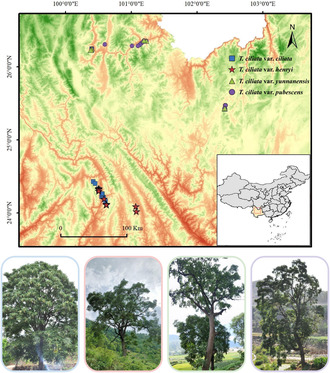

## 1 INTRODUCTION

*Toona ciliata*, aka Chinese mahogany (Edmonds & Staniforth, 1998), belongs to the *Toona* genus of the Meliaceae family and is an important timber species because of its high‐quality wood for industrial purposes in China (Chen et al., 1997). The species is naturally distributed in India, Malaysia, Indonesia, and other tropical and subtropical regions. It grows in the low‐altitude gully forests or in the hillside sparse forests in central and southern China, ranging from 300 to 2600 m above sea level. The species has been over exploited and was listed as an endangered species at Level II in China (Fu & Jin, 1992) although not listed as the species at risk by International Union for Conservation of Nature and Natural Resources (IUCN). Population density in natural forests declines due to the low natural regeneration (Liang et al., 2011) and potential inbreeding depression (Zhou et al., 2020). Because of these reasons, an important issue is concerned with genetic conservation of this endangered species in China. Genetic breeding programs have been set up by the state to improve wood quality and resistance to insects.

Another issue relevant to genetic conservation is concerned with intraspecific variation of *T. ciliata*. Currently, five varieties were recognized according to the leaf (size, shape, and pubescence) and flower (petal length and shape) traits (Chen et al., 1997). They are *T. ciliata* var. *ciliata*, *T. ciliata* var. *yunnanensis*, *T. ciliata* var. *pubescens*, *T. ciliata* var. *sublaxiflora*, and *T. ciliata* var. *henryi* (Appendix Table S1). These varieties are sympatry or partially overlapped in natural geographic distribution in Yunnan Province in China (Zhang, 2018), and natural hybridization among them cannot be excluded. However, morphological traits are often continuously distributed or poor for subdivision of intra‐ and interspecific variations (Ferreira et al., 2021), which makes it difficult to delimit varieties from morphological traits. Previous genetic studies mainly referred to the *T. ciliata* complex, without identifying specific varieties. These include provenance trials (Li et al., 2017; Wen et al., 2012), population genetic variation assayed by sequence‐related amplified polymorphisms (SRAP) and simple sequence repeat (SSR) markers (Li et al., 2015; Zhan et al., 2019), chloroplast DNA (cpDNA) markers (Hu, 2019), and nuclear internal transcribed spacer (ITS) and mitochondrial DNA markers (Xiao et al., 2023). Few studies investigated genetic variation in a specific variety except for *T. ciliata* var. *pubescensis* assayed by SSR markers (Liu et al., 2009). All these previous studies provided a general picture of genetic variation in the *T. ciliata* complex.

Classification of varieties consequently complicates genetic conservation of *T. ciliata* (Andriamihaja et al., 2020; Ennos et al., 2005; Panitsa et al., 2011). This is because genetic conservation in terms of single variety neglects natural hybrids among varieties in the sympatric region and species interactions. Whether genetic conservation is based on the *T. ciliata* complex or on single variety remains uncertain. Therefore, it is of practical significance to clarify these concerns for genetic conservation of this endangered species in China (Federici et al., 2013).

In this study, we examined the phylogenetic relationship of four varieties of *T. ciliata* at a fine spatial scale based on previous studies (Xiao et al., 2023; Zhang, 2018). All these varieties occur in Yunnan Province and are sympatric in geographical distribution (Chen et al., 1997; Zhang, 2018). The similarity and differences among these varieties provide important information to genetic conservation. It also aids in our understanding the mechanisms of sympatric speciation at the fine scale (Xiao et al., 2022). Here, we reported the phylogenetic relationships and the genetic differentiation among sympatric varieties with both the whole chloroplast genome sequence and markers, and explored the conservation strategy of this endangered species. Analysis with nuclear genomes will be present in a separate study using our recent assembly of nuclear genome sequences of *T. ciliata* as the reference genome (Wang, Xiao, He, Li, Lv, et al., 2022; Wang, Xiao, He, Li, Song, et al., 2022). One reviewer suggested inclusion of nuclear data analyses, but it is more appropriate to present the nuclear part in a separate study to accompany this work since more extensive analyses are needed with the resequencing data of these varieties.

It is well‐known that chloroplast genome has multiple attributes for phylogenetic and population structure analysis (Hu et al., 2019; Ravi et al., 2008), including (i) uniparental inheritance (maternal inheritance in most angiosperms but paternal inheritance in most gymnosperms), (ii) a relatively small genome size (120–220 kb) (Palmer, 1985), and (iii) a low mutation rate compared with the mutation rate of nuclear genomes (Chmielewski et al., 2015; Provan et al., 2001). The chloroplast genome sequences or markers have been widely applied to assessing both phylogeny and population genetic variation (Hu et al., 2019). Although discordance was reported in phylogeny in terms of organelle genomes versus morphological traits in the literature (Brown et al., 2010; Ebersbach et al., 2020), this remains uncertain regarding the phylogenetic relationship of varieties of *T. ciliata* (Leliaert et al., 2009).

We addressed the following questions: (1) Could the whole chloroplast genome sequences resolve the morphology‐based delimitation of varieties of *T. ciliata*? (2) How genetic variation was distributed within and between sympatric varieties of *T. ciliata* in terms of chloroplast genomes? To answer the first question, we conducted phylogenetic analyses among four sympatric varieties using the whole chloroplast genome sequences and compared the results with morphologic delimitation. To answer the second question, we conducted comparative genomic analyses among varieties using the whole chloroplast genome sequences and investigated genetic differentiation with expanded samples using two markers at the fine scale. To derive a more general conclusion assayed by markers, we employed one marker in coding region (*psa*A) and the other in noncoding region (*trn*L‐*trn*F) region. Besides, we evaluated the potential effects of natural selection on shaping chloroplast genomic divergence among sympatric varieties. The overall results were then synthesized to explore an appropriate strategy for genetic conservation of *T. ciliata*.

## 2 MATERIALS AND METHODS

### 2.1 Taxonomic sampling and DNA extraction

**FIGURE 1:**
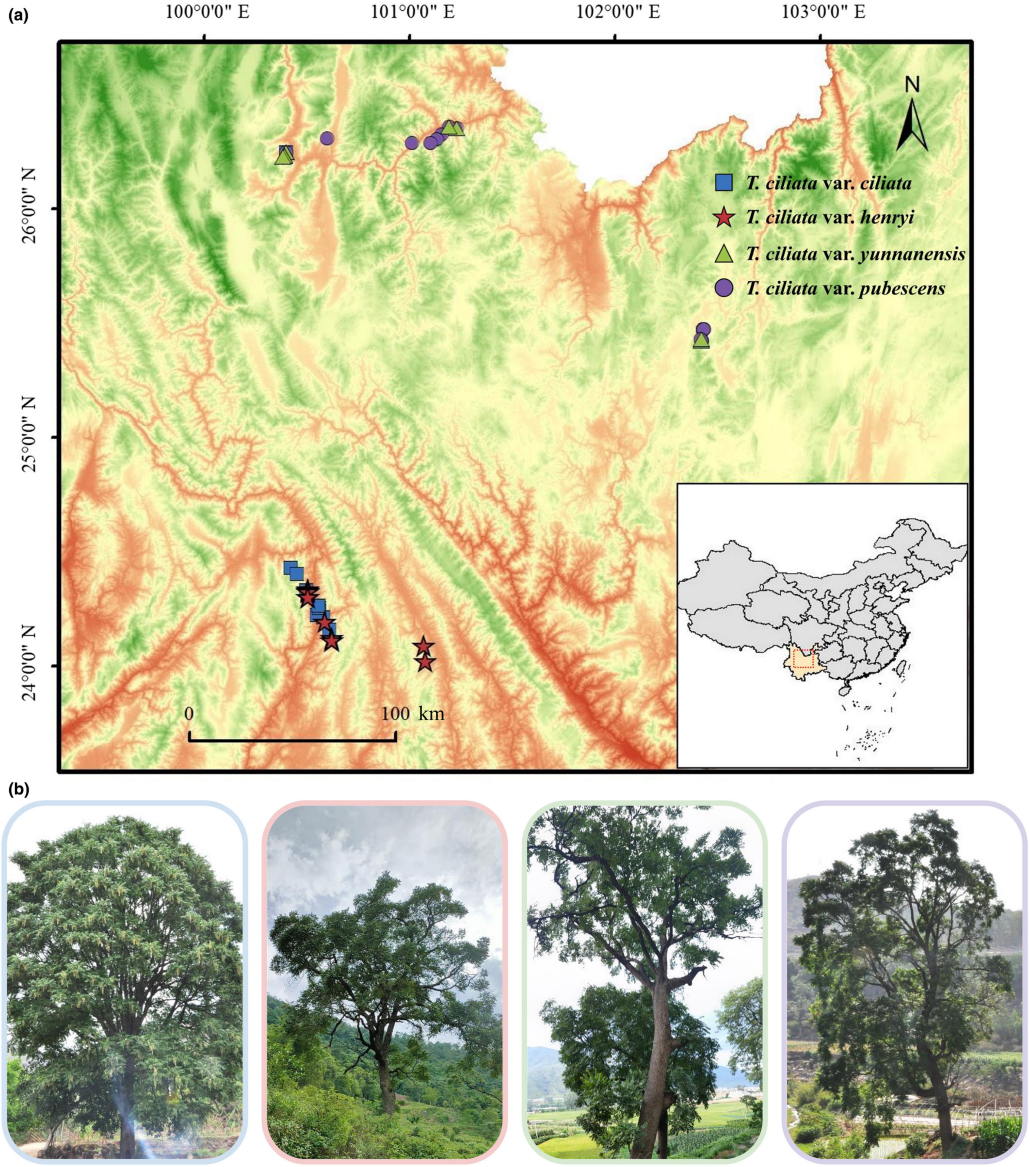
Sample sites and images of four varieties of *T. ciliata* in Yunnan Province: (a) Sampling sites of 58 individuals. The four varieties are *T. ciliata* var. *ciliata* in blue, *T. ciliata* var. *henryi* in red, *T. ciliata* var. *yunnanensis* in green, and *T. ciliata* var. *pubescens* in purple. (b) The bottom four tree images from left to right are *T. ciliata* var. *ciliata*, *T. ciliata* var. *henryi*, *T. ciliata* var. *yunnanensis*, and *T. ciliata* var. *pubescens*, respectively.

In view of a previous study on varieties of *T. ciliata* (Zhang, 2018), we collected leaf samples of varieties in different locations in Yunnan Province of China (Figure 1a). These samples were taxonomically identified according to both the field observations and literature records (Chen et al., 1997; Zhang, 2018). Appendix Table S1 provides detailed information of the specimens of these four varieties with voucher numbers. We collected 12 samples of *T. ciliata* var. *ciliata* in Puer and Lijiang cities, 13 samples of *T. ciliata* var. *henryi* in Puer city, 20 samples of *T. ciliata* var. *pubescens*, and 13 samples of *T. ciliata* var. *yunnanensis* in Lijiang and Kunming cities (Appendix Table S2). Note that *T. ciliata* var. *sublaxiflora* was not found in natural forests and hence was not included in this study. Figure 1b provides an image of an adult tree for each variety growing in Yunnan Province. All collected samples were located at more than 100 meters away among individuals to avoid genetic relatedness in natural forests. For whole chloroplast genome sequencing, we randomly selected three samples of each variety (Appendix Table S2). To investigate population genetic differentiation at the fine spatial scale, we analyzed all 58 samples with two markers. The sampling had been allowed by the Chinese Government and complied with the laws of the People's Republic of China. The fresh sample leaves were immediately dried with silica gel and then transported to laboratory for DNA extraction.

Total genomic DNA of each sample was extracted using CTAB method (Doyle & Doyle, 1987). The quality of extracted DNA was checked by following analyses: (i) use of 0.8% agarose electrophoresis to detect DNA samples for degradation and impurity, and to estimate the DNA concentration; (ii) use of Nanodrop Spectrophotometer (Thermo Scientific) to detect the concentration and purity of samples; and (iii) use of Qubit 2.0 Fluorometer (Life Technologies) to detect the concentration of samples.

### 2.2 Library construction and high‐throughput sequencing

Three individuals of each variety were sequenced by Science Corporation of Gene (SCGene), Guangzhou. Tested samples were made according to the standard procedure of IllumianDNA library, and a double‐end sequencing library with an insert size of 350 bp was constructed. After the construction of DNA library, qPCR method and Alignment 2100 Bioanalyzer (Alilent Technologies) were used for quality control. The DNA library that passed the quality inspection was sequenced by Illumina Novaseq 6000 (Illumination) high‐throughput sequencing platform. The sequencing strategy was pair‐end 150, and the sequencing data were not <1 Gb.

Illumina high‐throughput sequencing results were originally presented as raw image data files, which were converted to raw reads after base calling by the CASAVA software. Raw reads from Illumina sequencing were subjected to adaptor trimming and filtering of low‐quality reads by fastp v0.20.1 (https://github.com/OpenGene/fastp; Chen et al., 2018). The minimum length for reads after trimming was set to 150 nucleotides, and the quality threshold was set to Q20. Details for quality control were summarized in Appendix Table S3.

### 2.3 Chloroplast genome assembly and annotations

With the clean reads after trimming (Appendix Table S3), genome sequences were de novo assembled using SPAdes v.3.15.2 (http://cab.spbu.ru/publication/new‐frontiers‐of‐genome‐assembly‐with‐spades‐3‐0/), with k‐mer sizes of 33, 55, 79, 97, and 127. The assembled contigs included a mixture of sequences from organellar and nuclear genomes. We identified chloroplast contigs using similarity searches by BLASTN 2.13.0+ against NCBI nucleotide collection (nt) database. We had assessed the assembly quality of these chloroplast genomes by mapping raw paired‐end reads to the chloroplast genome using bowtie2 (v2.3.5.1; Langmead & Salzberg, 2012) and generated sequencing depth and coverage map according to Ni's protocol (Ni et al., 2023).

Genes were annotated using CpGAVAS (Liu et al., 2012) and ORF Finder (https://www.ncbi.nlm.nih.gov/orffinder/). For the preliminary results of annotations, the accuracy of the results was verified by comparing the encoded proteins and rRNA with the reported chloroplast genome of *T. ciliata* (GenBank access no.: NC_039592) using Blastn 2.13.0 (https://blast.ncbi.nlm.nih.gov/Blast.cgi) against NCBI nucleotide collection (nt) database. All proteins had been verified by using similarity searches by Blastp 2.13.0 against NCBI nonredundant protein sequences (nr) database (Zhang et al., 2000). ARWEN (Laslett & Canbäck, 2008) was used to annotate tRNA. If abnormal tRNA occurred, the verification would be carried out again in combination with tRNAscan‐SE 2.0 predictions (Lowe & Chan, 2016). The gene map of chloroplast genome was drawn using OGDRAW (Greiner et al., 2019). The final genome sequences of all 12 samples were deposited in GenBank under the accession numbers OM135324–OM135327 and OP373439–OP373446.

### 2.4 Comparative genome analysis among varieties

Alignments of chloroplast genomes of the four varieties were conducted using ClustalW from MEGAX (Kumar et al., 2018) with default parameters. Tandem repeat finder (TRF) (Benson, 1999) and microsatellite identification tool (MISA v2.1) (Beier et al., 2017) were used to search for repetitive sequences. To visualize the variation among the 12 samples, we analyzed the homology of their genome sequences using mVISTA (Frazer et al., 2004) where the sequence of *T. ciliata* (GenBank access no.: NC_039592) was used as the reference. The nucleotide diversity per site (*π*) was estimated using DnaSP v5 (Librado & Rozas, 2009).

### 2.5 Phylogenetic analysis and divergent time estimation

To evaluate the phylogenetic relationship among varieties, we selected *T. sinensis* (GenBank access no.: MW401816) and *Melia azedarach* (access no.: NC_050650) of the same family (Meliaceae) as outgroups. To derive a reliable tree, we used the concatenated whole genome sequences that were orthologous among six taxa and aligned for phylogenetic analysis using clustalW algorithm in MEGAX (Kumar et al., 2018). Note that only one IR region was included. The general time reversible (GTR) model with a discrete Gamma distribution (GTR + G) was detected as the best‐fit‐model according to Akaike information criterion (AIC) and Bayesian information criterion (BIC). We constructed a maximum likelihood (ML) tree using MEGAX with 1000 Bootstrap replications and the GTR + G model.

BEAST v1.10.4 (Suchard et al., 2018) was used to reconstruct phylogenetic relationship and estimate divergent times, with a Yule process as the prior, the assumption of an uncorrelated lognormal relaxed clock, and the GTR + G model. Three calibration times were selected for estimating divergent times. The divergent time between *Toona* and *Melia* was approximately 68.3 Mya with a 95% HPD of 80.5–52.8 Mya (Appelhans et al., 2012). For calibrating branch ages containing all members of the *Toona* and *Melia* genera, the mean age of the most recent common ancestor (MRCA) was set at 68.3 Mya, with a prior normal distribution and a standard deviation of 7.1 Mya. The age of the MRCA between *T. sinensis* and *M. azedarach* was set at 48.0 Mya, with a standard deviation of 0.3 and a 95% confidence interval [47.8, 48.9] Mya from the TimeTree database (Grudinski et al., 2014; Kumar et al., 2017; Li, Yi, et al., 2019). The divergent time between *T. ciliata* and *T. sinensis* was set at 31.6 Mya, with a standard deviation of 8.0 and a 95% confidence interval [15.1, 46.5] Mya (Muellner et al., 2010).

The length of Markov chain Monte Carlo (MCMC) was set as 5.0 × 10^8^ generations and data were sampled every 1000 generations. Effective sample size (ESS) was checked in Tracer v1.7.2 (Suchard et al., 2018) and accepted for further analysis under a large ESS (>200). We used TreeAnnotator v1.10.4 to build the maximum clade credibility (MCC) tree with the first 20% of samples as the burn‐in, and the tree derived from Bayesian inference (BI) was compared with the ML tree derived from MEGAX under GTR + G model. The phylogenetic trees were graphed using iTOL (https://itol.embl.de).

### 2.6 Detection of positive and purifying selection

Both branch‐ and site‐models with codeml from PAML v4.9 package were used to detect selection in terms of the ratio of nonsynonymous to synonymous substitutions (*ω* = *dN*/*dS*) for all protein‐coding genes (Yang, 2007; Yang & Bielawski, 2000). The branch models with one‐*ω* versus two‐*ω* ratios were tested using likelihood ratio test (LRT): 2∆ℓ = 2log(*L*_1_‐*L*_0_), where *L*_1_ is the log‐likelihood under the alternative hypothesis (i.e., the two‐*ω* ratios model) and *L*_0_ is the log‐likelihood under the null hypothesis (i.e., the one‐*ω* model). The statistics 2∆ℓ follows a chi‐squared distribution with the degree of freedom being equal to the different number of parameters between null and alternative hypotheses. The branch for each of the four varieties was separately set as the foreground branch and the remaining branches as the background branches (Figure S1), with the aim at detecting selection in each variety.

The site model was separately applied to analyzing the concatenated sequences of protein‐coding genes in large single copy (LSC), small single copy (SSC), inverted repeat (IR), and the whole genome regions with one IR included. Two pairs of contrasting site models, M2a (selection) versus M1a (neutral), M8 (beta & *ω* > 1) versus M7 (beta), were used to detect positive selection sites. Likelihood ratio test was used to test the significance of alternative models. Posterior probabilities at each amino acid site were estimated from three site classes under M2a model using naïve empirical Bayes (NEB) procedure, and a probability of not ≤0.95 indicated a positive selection site (Yang & Swanson, 2002).

### 2.7 Genetic differentiation among varieties

To investigate population differentiation at the fine scale, we found single nucleotide polymorphisms (SNPs) from the alignment of whole chloroplast genome sequences among the four varieties and designed a pair of primers with polymorphisms in *psa*A region. The designed primers were 5′‐ATCGCCGTGTTGTAACAGAGA‐3′ and 5′‐TGATTCTTCCTGGGTCGATGC‐3′. From the literature (Taberlet et al., 1991), we selected a polymorphic marker in the intergenic region *trn*L‐*trn*F, and the designed primers were 5′‐CGAAATCGGTAGACG CTACG‐3′ and 5′‐ATTTGAACTGGTGACACGAG‐3′. The PCR was performed in 25 μL volume, which contained 1 μL plant DNA, 12.5 μL 2 × Es Taq MasterMix (0.1 U Tap polymerase, 500 μmol/L dNTPs, 20 mmol/L Tris–HCl, 3 mmol/L MgCl_2_, 100 mmol/L KCl), 1 μL of each primer, and 9.5 μL ddH_2_O. The PCR protocol was set below: preheating at 95°C for 4 min, 35 cycles at 94°C for 30 s, annealing at 55°C for 1 min, and elongation at 72°C for 1 min, followed by a final extension at 72°C for 10 min. The PCR products were subjected to agarose gel electrophoresis at 2%, and the results were detected by gel imager. The single amplified product with clear bands was sent to Sangon Biotech (Shanghai) Co., Ltd for sequencing.

The multiple sequence alignment was conducted with ClustalW algorithm from MEGAX with default parameter settings. The sequences of 363 bp for *psa*A and 969 bp for *trn*L‐*trn*F were used for downstream analyses, and the aligned sequences were submitted to figshare (https://doi.org/10.6084/m9.figshare.24203763). To explore genetic variation among four varieties, analysis of molecular variance (AMOVA) was done separately for sequences of *psa*A, *trn*L‐*trn*F and their concatenated sequences (*psa*A‐*trn*L‐*trn*F) using Arlequin v3.0 (Excoffier et al., 2005).

Genetic differentiation among varieties was also analyzed by comparing population differentiation in terms of marker sequence divergence (*N*_st_) versus in terms of allele frequency (*F*_st_) (Pons & Petit, 1996). DnaSP v6 (Rozas et al., 2017) was used to estimate *N*_st_ and *F*_st_. A difference between *F*_st_ and *N*_st_ was tested using permutations (Rozas et al., 2017). All these estimates were obtained using the concatenated sequences of these two markers.

Isolation by distance (IBD) at the fine scale was tested using the regression of *F*_st_/(1−*F*_st_) on the logarithm of geographic distance (Rousset, 1997). Pairwise population differentiation (*F*_st_) was calculated by Arlequin v3.0 (Excoffier et al., 2005) using the concatenated sequences. Geographical distance matrix among pairwise varieties was calculated from central sampling sites (*T. ciliata* var. *ciliata*: 26°24′19″ N, 100°39′17″ E; *T. ciliata* var. *henryi*: 24°32′60″ N, 100°47′39″ E; *T. ciliata* var. *yunnanensis*: 25°38′43″ N, 102°38′54″ E; *T. ciliata* var. *pubescens*: 25°43′10″ N, 102°39′34″ E).

## 3 RESULTS

### 3.1 Genome sequences of four varieties

**FIGURE 2:**
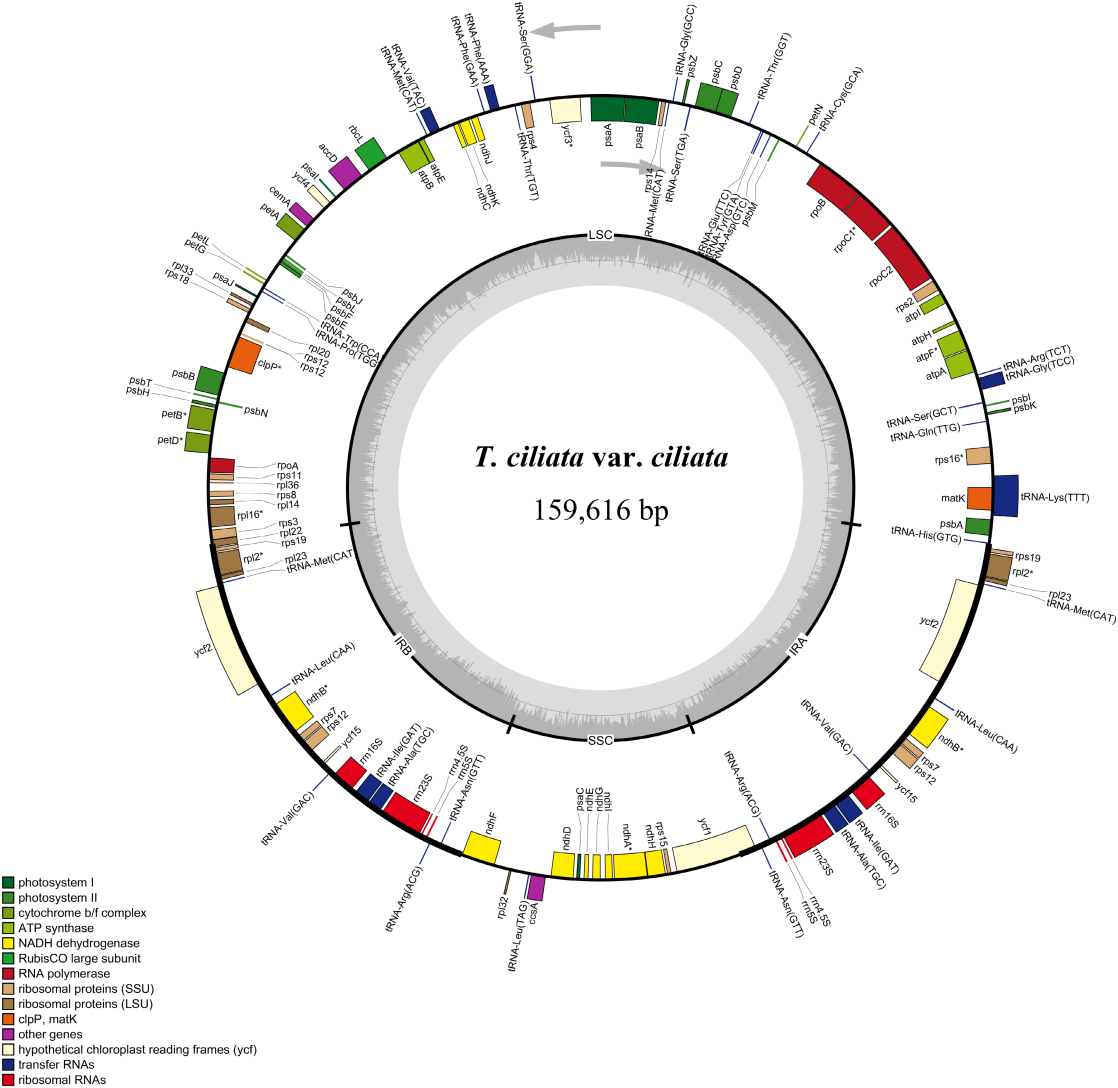
Circular map of the de novo assembled chloroplast genome of *T. ciliata* var. *ciliata* chloroplast genome. Gray arrows indicate the direction of gene transcription. The genes inside and outside the circle are transcribed in clockwise and counterclockwise directions, respectively. Genes in different functional groups are shown in different colors. The darker gray columns in the inner circle correspond to GC content. Regions of small single copy (SSC), large single copy (LSC), and inverted repeats (IRA, IRB) are indicated.

A high‐quality assembly of chloroplast genome was obtained for each variety. Details for genome assembly information were provided in Appendix Table S3. For instance, the Q30 values ranged from 89.8% to 93.4% and the average sequencing depth was >180×. The coverage rate was 100% for all sequencing of 12 samples. The complete sequences of 12 samples were reposited to NCBI GenBank (access numbers: OM135324–OM135327 and OP373439–OP373446). Their genome sizes ranged from 159,546 bp to 159,617 bp in length. There were 334 SNPs and 96 indels among the 12 samples. The genome exhibited a circular molecule with typical quadripartite structure of angiosperms (LSC, SSC, IRA, and IRB), and harbored 103 genes in total, including 79 protein‐coding genes, 20 tRNA genes, and 4 rRNA genes. Figure 2 shows the circular chloroplast genome map of *T. ciliata* var. *ciliata*, and the remaining three varieties had a similar genome structure except for small differences in size due to low mutation rates.

A comparison indicated that highly conservative chloroplast genomes occurred among the four varieties (Table 1). LSC and SSC regions exhibited relatively higher divergence than IR regions. The SSC region had the largest nucleotide diversity per site (*π* = 0.00039), followed by the LSC (*π* = 0.00036) and IR regions (*π* = 0.00004). A similar pattern of nucleotide diversity per site was also observed for the protein‐coding sequences in SSC (0.00037), LSC (0.00035), and IR (0.00023) regions. A global comparison of chloroplast genome homology across the 12 samples showed high sequence similarity and indicated that the chloroplast genomes of the four varieties were highly conserved (Appendix Figure S2).

**TABLE 1:** Polymorphic sites and nucleotide diversity among four varieties of *Toona ciliata.*

Region	Coding and noncoding sequences			Protein‐coding sequences		
	Site	Polymorphic site	*π*	Site	Polymorphic site	*π*
LSC	87,234	108	0.00036	43,422	46	0.00035
SSC	18,423	25	0.00039	14,271	17	0.00037
IR	27,066	4	0.00004	10,419	6	0.00023
LSC, IR, and SSC	132,723	137	0.00030	68,112	69	0.00034

**FIGURE 3:**
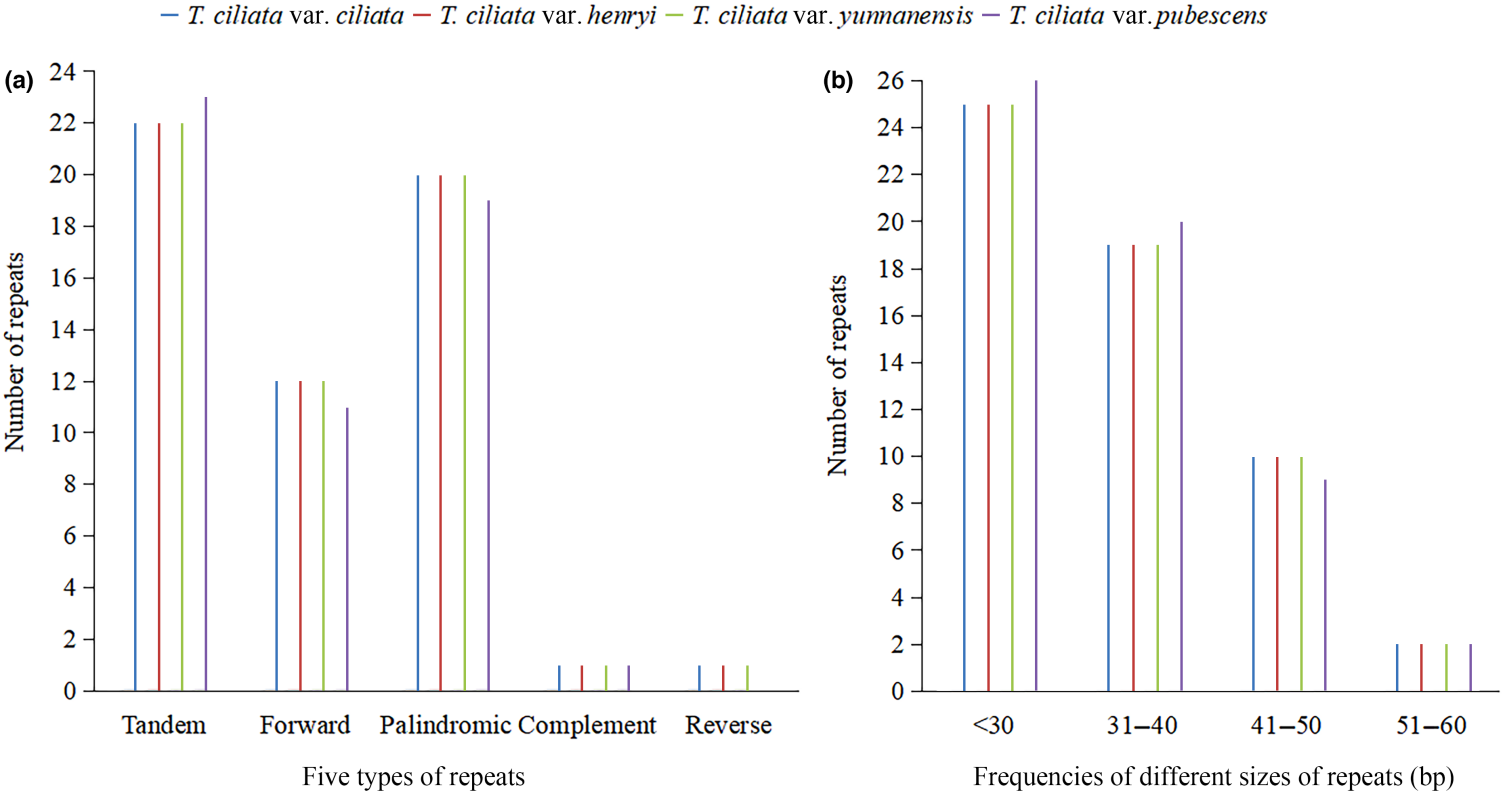
Comparison of repetitive sequences among four varieties: (a) Five types of repeats; (b) Frequencies of different sizes of repeats (bp).

Sequence repeats, including tandem, forward, palindromic, complement, and reverse repeats, were identified. Figure 3 shows that these repeats were generally conservative among the four varieties. There were 54–56 repetitive sequences in total where 11–12 forward repeats and 19–20 palindromic repeats were separately detected. Tandem repeat sequences occurred most frequently, with 22 in the cpDNA sequences of *T. ciliata* var. *ciliata*, *T. ciliata* var. *henryi*, and *T. ciliata* var. *yunnanensis*, and 23 in the cpDNA sequence of *T. ciliata* var. *pubescens*. Few complementary and reverse repeats were observed (Figure 3a).

The repetitive sequences in the four varieties were between 30 and 58 bp (Figure 3b). Most repeats were distributed in noncoding regions (the intergenic regions and introns), and only a few in coding genes. For instance, there were seven repeated sequences in *ycf*2 gene, but only one repeat in *psa*B, *psa*A, and *ndh*F genes (Appendix Tables S4 and S5).

Table 2 summarizes different types of SSRs in the four varieties. *T. ciliata* var. *pubescens* had 244 SSRs, and the remaining three varieties had 243 SSRs. The predominant type of SSRs was mononucleotide repeats, with A/T accounting for 74.07% of SSRs in *T. ciliata* var. *ciliata*, *T. ciliata* var. *henryi* and *T. ciliata* var. *yunnanensis*, and 73.77% in *T. ciliata* var. *pubescens* (Appendix Figure S3). Other types of SSRs accounted for a small proportion, with a decreasing abundance from dinucleotides to tetranucleotides, to trinucleotides, and to pentanucleotides. The chloroplast genome of *T. ciliata* var. *pubescens* had one unique hexanucleotide SSR that was absent in the genomes of the remaining three varieties.

**TABLE 2:** Distribution of SSRs of chloroplast genomes in four varieties of *Toona ciliata.*

Taxon		*T. ciliata* var. *ciliata*	*T. ciliata* var. *henryi*	*T. ciliata* var. *yunnanensis*	*T. ciliata* var. *pubescens*
Genome Size (bp)		159,617	159,617	159,615	159,550
SSR type	Mononucleotide	184	184	184	184
	Dinucleotide	42	42	42	41
	Trinucleotide	5	5	5	5
	Tetranucleotide	11	11	11	11
	Pentanucleotide	1	1	1	2
	Hexanucleotide	0	0	0	1
	Total	243	243	243	244
Protein‐coding sequence	No.	56	56	56	57
	%	23.05	23.05	23.05	23.36

A few coding genes, including *rpo*C2, *rbc*L, *pet*A, *ycf*2, *ndh*F, *ndh*D, *ycf*1, and *ycf*2, contained more than two types of SSRs in *T. ciliata* var. *ciliata*, *T. ciliata* var. *henryi*, and *T. ciliata* var. *yunnanensis*. The coding sequences had 23.05% of the total number of SSRs in these three varieties, but 23.36% in *T. ciliata* var. *pubescens*. In addition, a hexanucleotide SSR occurred in gene *rpo*A of *T. ciliata* var. *pubescens*.

### 3.2 Phylogenetic relationships among varieties

**FIGURE 4:**
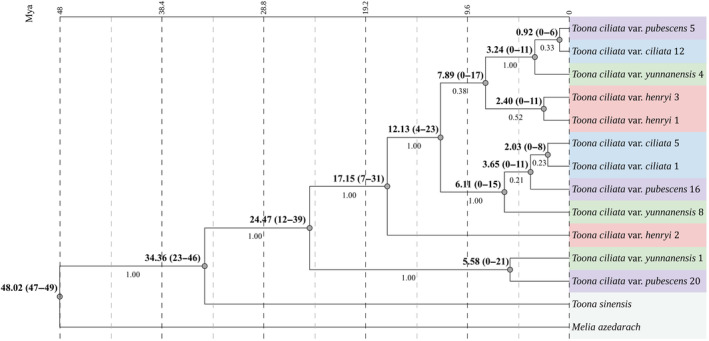
Phylogenetic relationship among varieties derived from the whole chloroplast genome under the model of relaxed molecular clock and uncorrelated rates. The bold value at each node represents mean age (Mya), and the values in parentheses are the 95% HPD intervals around point age estimates. Branch labels represent Bayesian posterior probabilities.

The topology of maximum clade credibility (MCC) tree derived from Bayesian inference (BI) generally matched the ML tree using the concatenated sequences of LSC, SSC, and one IR regions. Figure 4 shows that samples of the four varieties were not monophyletic in MCC tree. Appendix Figure S4 provides the ML tree constructed by using the whole‐genome sequences with a single IR region. For instance, the clade of *T. ciliata* var. *yunnanensis* (the first sample) and *T. ciliata* var. *pubescens* (the twentieth sample) had a Bayesian posterior probability of 100%. The remaining subclades had lower Bayesian posterior probabilities, ranging from 21% to 52% (Figure 4).

The MCC tree derived from BEAST analysis provided the point estimates of ages and 95% HPD (Figure 4). The divergent time between *M. azedarach* and genus *Toona* was estimated as 48.02–34.36 Mya. The largest divergent time among varieties was approximately 24.47 Mya (95% HPD: 39–12 Mya). The most recent divergent time was about 0.92 Mya (95% HPD: 0–6 Mya).

### 3.3 Test of purifying and positive selections

Natural selection was tested in 79 polymorphic protein‐coding genes. Likelihood ratio tests showed that the two‐ω‐ratios model was not significantly different from the one‐ω‐ratio model (Appendix Table S6), indicating that the *ω* estimates were essentially consistent among the four varieties.

We removed 21 genes whose *dN* or *dS* values were close to 0, which otherwise yielded overflow or zero estimates of *dN/dS* ratios. These genes were more conservative among varieties. The remaining 58 protein‐coding genes were used to detect natural selection based on the phylogeny of the four varieties (Appendix Table S6).

**FIGURE 5:**
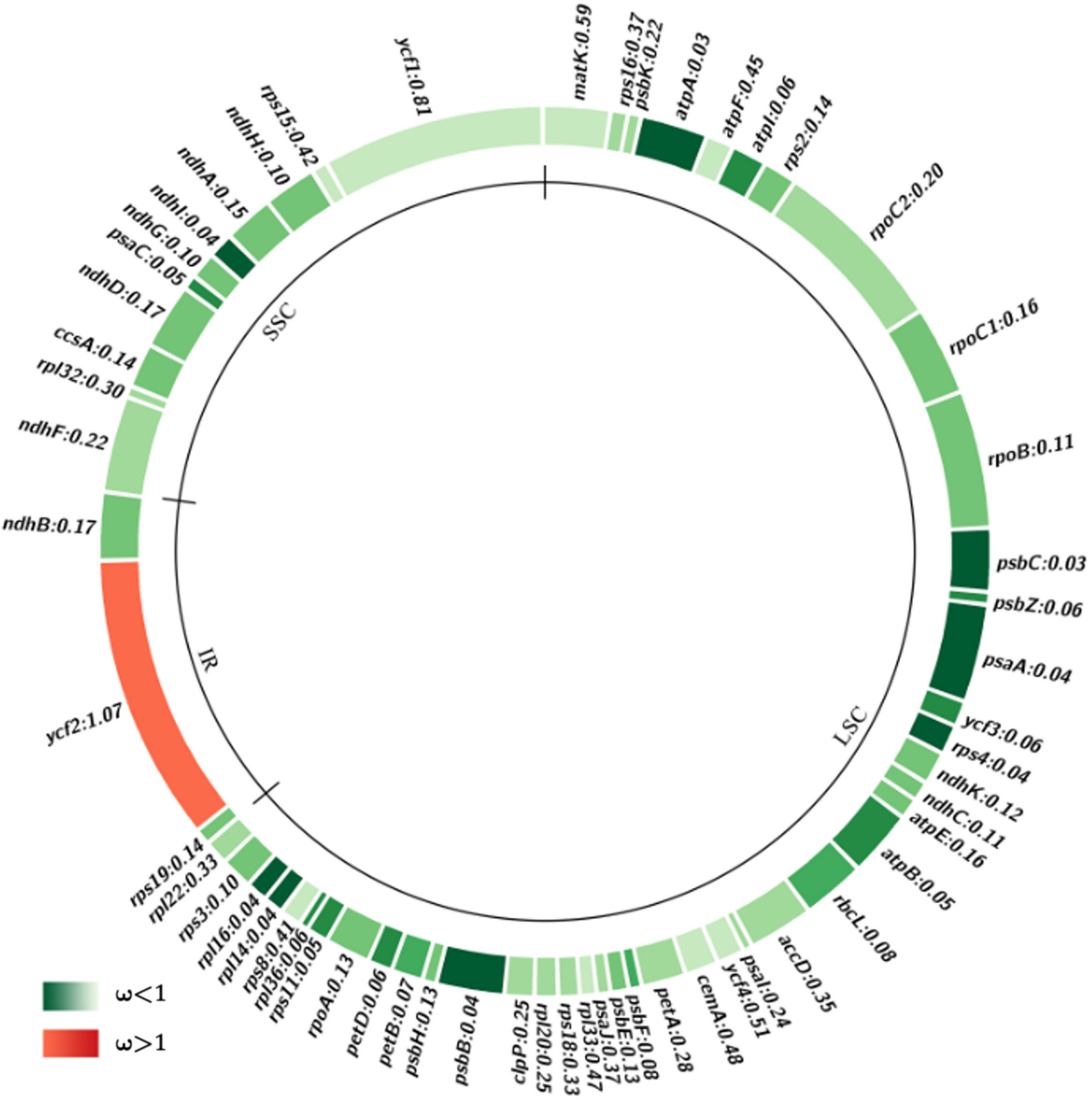
Estimates of *dN/dS* of 58 protein‐coding genes in four varieties of *Toona ciliata*.

Figure 5 shows that most protein‐coding genes were under strong purifying selection. Weak positive selection was present only in *ycf*2 gene (*ω* = 1.07). Pairwise analysis with yn00 from PAML package between each of the four varieties of *T. ciliata* with *T. sinensis* or with *M. azedarach* also confirmed positive selection in *ycf*2 gene (data not shown here). Genes in IR region had lower *ω* values (*ω* = 0.1540 ± 0.0263), whereas genes in LSC and SSC regions had relatively higher *ω* values (*ω* = 0.1889 ± 0.1578 in LSC region, and *ω* = 0.2282 ± 0.2243 in SSC region).

Random‐site models were applied to mapping amino acid sites under positive selection (Yang, 2006). The naïve empirical Bayes procedure was used to calculate the posterior probabilities of positive selection (*ω* > 1) under different model assumptions. Appendix Table S7 shows the LRTs of positive selection in LSC, SSC, and IR regions.

In the LSC region (60 genes from *rps*12 to *rpl*22 in genome, Figure 2), LRT between M2a and M1a models was 2Δl = 7.2247 (Appendix Table S8), greater than $\chi_{0.05,\mathrm{df}=2}^{2}$ = 5.9914, indicating the presence of positive selection. Test with M8 (beta & *ω* > 1) versus M7 (beta) also indicated the presence of positive selection in some amino acid sites (*p*‐value = .0063; Appendix Table S7). The *rpo*C2 gene had two amino acid sites with positive selection, 3212 V (valine) and 3772 Q (glutamine), while genes of *cem*A, *rps*2, *rps*8 and *rpl*22 had only one amino acid site with positive selection, 2314 S (serine), 10,893 H (histidine), 13,713 L (leucine) and 14,414 P (proline), respectively (Appendix Table S7).

In the IRA region (7 genes from *rps*19 to *ycf*15 in genome, Figure 2), LRTs with M2a versus M1a or M8 versus M7 showed the presence of positive selection (*p*‐value ~10^−9^; Appendix Table S8). There was one amino acid site under positive selection in the *rpl*23 gene, 14,861 G (glycine), and four in the *ycf*2 gene, including 16,183 N (asparagine), 16,585 R (arginine), 16,589 T (threonine), and 16,591 L (Appendix Table S7).

In the SSC region (12 genes from *ndh*F to *ycf*1 in genome, Figure 2), LRTs with M2a versus M1a or M8 versus M7 showed significant differences (*p*‐value ~10^−9^; Appendix Table S8), indicating the presence of positive selection in some amino acid sites. Two sites under positive selection were detected only in *ycf*1 gene, 21,308 G and 21,468 T (Appendix Table S7).

Compared with the results in Appendix Table S7, LRTs with the catenated sequences of all 79 protein‐coding genes exhibited a comparable number of sites under positive selection (Appendix Table S9). For instance, estimates with M2a were *ω*_0_ = 0.1138, *ω*_1_ = 1.0000, and *ω*_2_ = 13.3938, with proportions of *p*_0_ = 0.9046, *p*_1_ = 0.0856, and *p*_2_ = 0.0098. LRT with M2a versus M1a showed that 12 sites were under positive selection (Appendix Table S8). Appendix Figure S5 shows the distribution of amino acid sites under positive selection. Most of these amino acid sites were consistent with the preceding results derived from analyses in individual segments (LSC, SSC, and IR; Appendix Table S7). For instance, compared with the analysis of the SSC region only, more positive selection sites in *ycf*1 were detected with the concatenated sequences of 79 coding genes, including 21,225 T, 21308 G, 21468 T, 21505 E (glutamic acid), 21,529 K (lysine), 21,862 S, and 22,440 T. The *ndh*F gene had only one site under positive selection, 18,391 H. There were two sites under positive selection in the *ycf*2 gene in IR region, 16,183 N and 16,589 T. Both the *rpo*C2 and *rps*8 genes in the LSC region had only one positive selection site, 3772 Q and 13,713 L, respectively. Reasons for such differences could arise from offsetting effects among positive and negative amino acid sites when different lengths of genome segments were analyzed.

### 3.4 Genetic differentiation among varieties

Sequences of two markers (*psa*A and *trn*L‐*trn*F) were analyzed using AMOVA. The results indicated that most variation was within varieties: *Ф*_st_ = 0.0569, *p*‐value = .0166 for the *psa*A region; *Ф*_st_ = 0.0310, *p*‐value = .0675 for the *trn*L‐*trn*F region; and *Ф*_st_ = 0.0351, *p*‐value = .0430 for the concatenated sequences (Table 3). Generally, a low level and insignificant genetic differentiation occurred among varieties.

**TABLE 3:** Analysis of molecular variance (AMOVA) of chlorotypes among varieties of *Toona ciliata.*

cpDNA segment	Source of variation	df	Sum of square	Variance component	Percentage of variance (%)	*Ф*_st_	*p*‐Value
*psa*A	Among varieties	3	5.592	0.0605	5.69	0.0569	.0166
	Within varieties	54	54.097	1.0018	94.31		
	Total	57	59.690	1.0623			
*trn*L‐*trnF*	Among varieties	3	23.958	0.1754	3.10	0.0310	.0675
	Within varieties	54	296.128	5.4839	96.90		
	Total	57	320.086	5.6592			
*psa*A and *trn*L‐*trn*F	Among varieties	3	29.550	0.2359	3.51	0.0351	.0430
	Within varieties	54	350.226	6.4857	96.49		
	Total	57	379.776	6.7215			

**FIGURE 6:**
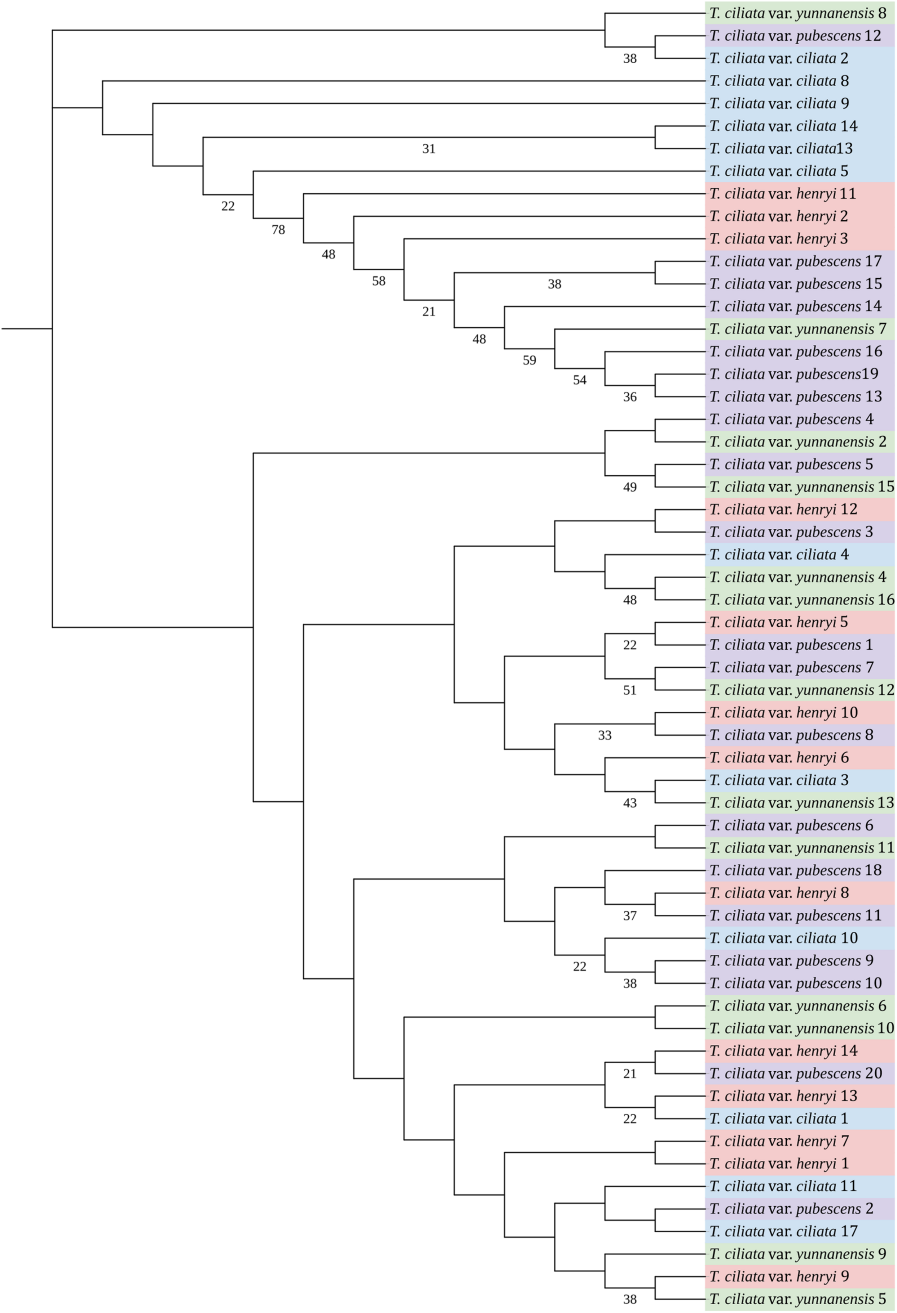
Phylogenetic relationship derived from the maximum likelihood (ML) analysis among 58 individuals of four varieties of *Toona ciliata* using the catenated sequences of *psa*A and *trn*L‐*trn*F markers. Branch labels represent bootstrap supporting values of >20%.

The maximum likelihood tree indicated that taxon samples were not grouped into distinct clusters in terms of the catenated sequences of these two markers, which was discordant with variety delimitation according to morphological traits (Figure 6).

Genetic differentiation of pairwise varieties was analyzed using the concatenated sequences of *psa*A and *trn*L‐*trn*F segments. *T. ciliata* var. *ciliata* differed from *T. ciliata* var. *henryi* with *Ф*_st_ = 0.0534 (*p*‐value = .0587), from *T. ciliata* var. *yunnanensis* with *Ф*_st_ = 0.0276 (.0841), and from *T. ciliata* var. *pubescens* with *Ф*_st_ = 0.0520 (.0782). *T. ciliata* var. *henryi* differed from *T. ciliata* var. *yunnanensis* with *Ф*_st_ = −0.0114 (.6764), and from *T. ciliata* var. *pubescens* with *Ф*_st_ = 0.0284 (.1730). Genetic differentiation was *Ф*_st_ = 0.0501 (.0596) between *T. ciliata* var. *yunnanensis* and *T. ciliata* var. *pubescens*. All pairwise analyses indicated insignificant genetic differentiation between varieties.

With the concatenated sequences (*psa*A‐*trn*L‐*trn*F), *N*_st_ (0.0348) was not significantly different from *F*_st_ (0.0354), indicating absent phylogeographic structure among varieties. Isolation by distance effects were not significant among varieties (*F*_st_/(1‐*F*_st_) = 0.0353–0.0074 ln(*distance*), *p*‐value = .2507).

## 4 DISCUSSION

In this study, we provided evidence that four varieties of *T. ciliata* were very closely related in terms of the variation of chloroplast genome sequences. This also provided evidence that a conflict between organelle genome‐ and morphology‐based delimitations in sympatric speciation, and implied distinct rates of lineage sorting processes between organelle and nuclear genomes (Hu et al., 2019). Although there was substantial population genetic differentiation in terms of the *T. ciliata* complex derived from nuclear markers (Xiao et al., 2023), the phylogenetic relationship among varieties in sympatric region was unknown at a fine scale. From the pattern of mitochondrial genome markers, Xiao et al. (2023) showed three distinct regions in natural distribution of the *T. ciliata* complex. This study investigated four sympatric varieties of *T. ciliata* in the eastern region (Zhang, 2018). The results partly consolidated the previous study on population genetic structure of the *T. ciliata* complex where varieties were not identified (Xiao et al., 2023).

### 4.1 Genome divergence and evolutionary process

The four varieties had the typical structure of angiosperm organelle genomes, with LSC, SSC, IRA, and IRB parts. Their genome sizes were comparable to those found in *T. ciliata*, *T. sinensis*, *T. sureni*, and other species in the Meliaceae family although the estimated numbers of genes were unequal among them (Lin et al., 2018; Mader et al., 2018; Tan et al., 2023; Xin et al., 2018). The four varieties exhibited small variation in chloroplast genome size, gene arrangement, and genomic structure. The nucleotide diversity per site (*π*) was much smaller compared with those of plant species in monophyly stage (Syring et al., 2007), such as the per‐site nucleotide diversity between species in *Lagerstroemia* and *Michelia* genera (Deng et al., 2020; Xu et al., 2017). However, the observed pattern of nucleotide diversity supported the commonality that the mutation rates are generally greater in LSC and SSC than in IR regions with repetitive properties (Li et al., 2016; Perry & Wolfe, 2002; Zhu et al., 2016). Generally, only a few mutations were accumulated in each region.

The repetitive sequences often exhibit large sequence variation among species in the monophyly stage (Ahmed et al., 2012). The four varieties showed that noncoding regions were less conservative than protein‐coding regions, consistent with previous reports (Wen et al., 2021). However, the same pattern of repetitive sequences in three varieties except for *T. ciliata* var. *pubescens* for a slight difference implicated that they were recently divergent.

Besides the low mutation rates, all protein‐coding genes except for *ycf*2 were under purifying selection (*ω* < 1). Chloroplast genomes accumulated deleterious mutations due to small effective population size (Kimura, 1962) and a lack of recombination. Strong purifying selection removed detrimental mutations in taxa. This also implied that the disruptive selection that fixed alternative alleles between varieties was effectively impeded at the gene level. Thus, purifying selection could play an important role in conserving sequence structure among varieties in sympatric region. Although there was positive selection only at a few amino acid sites, which could likely exhibit alternative gene expression, their joint effects were small. In addition, the pattern of average strength of purifying selection was consistent with the pattern of mutation rates in different regions (IR, LSC, and SSC).

### 4.2 Evolutionary divergence among varieties

The phylogenomic analysis implied that the four varieties were relatively recently divergent. Point estimates showed a wide range of divergent times (0.92–24.47 Mya) among varieties. Note that the large interval of the estimates of divergent times (Figure 4) could arise from the use of a wide range of divergent times derived from TimeTree database for calibrations (Kumar et al., 2017). This was the same situation for estimating the divergent times between *T. ciliata* and *T. sinensis* (Wang, Xiao, He, Li, Lv, Hu, et al., 2022; Wang, Xiao, He, Li, Song, et al., 2022). The fossil records are still lacking for the species that are closely related to the *Toona* genus. Nevertheless, our estimates of the divergent times between *M. azedarach* and genus *Toona* (48.02 Mya) or between *T. sinensis* and *T. ciliata* (34.36 Mya) were consistent with previous studies (Appelhans et al., 2012; Grudinski et al., 2014; Kumar et al., 2017; Li, Yi, et al., 2019).

Chloroplast genome markers are widely applied to analyzing both phylogeny and population structure (Hu et al., 2019; Xie et al., 2018). Markers in noncoding regions are commonly utilized for investigating genetic differentiation (Binks et al., 2021; Fang et al., 2010; Shaw et al., 2007), but there are also coding regions that are polymorphic and designed for markers, such as in *Saxifraga sinomontana* (Li et al., 2018), *Quercus liaotungensis* (Yang et al., 2018), and *Prunus armeniaca* (Li et al., 2021). Here, we discovered a polymorphic marker in *psa*A from sequence alignments, which is infrequently reported in other studies. The *rbc*L and *mat*K sequences are often used as genetic barcodes to identify species. In this study, polymorphic sites were absent in *rbc*L sequences among varieties, but present in *mat*K sequences only between *T. ciliata* var. *pubescens* and *T. ciliata* var. *yunnanensis*. The *rbc*L sequence is often used for identifying deep phylogenetic relationships due to the more conservative property of this gene. Both *rbc*L and *mat*K barcodes were not effective for identifying the four varieties. Analysis of genetic differentiation supported the mixture of genomic composition among the four varieties (Figure 6). We concluded that the morphological diversity among four varieties was not supported by the genetic divergence in terms of chloroplast genome sequences.

Incongruence cannot be ruled out between morphological‐ and organelle genome‐based species delimitations. Since the morphological traits (e.g., leaf and flower traits) are mainly controlled by nuclear genes, this incongruence could mainly arise from the cytonuclear phylogenetic conflict. Cytonuclear incongruence among closely related taxa is widely recorded in the literature. For instance, reports show mitochondrial‐nuclear discordance in phylogeny of animals, poplars, and other species (Funk & Omland, 2003; Huang et al., 2014; Sloan et al., 2017). Also, studies showed chloroplast‐nuclear discordance in phylogeny of different plant species (Hu et al., 2019; Lee‐Yaw et al., 2019; Mata‐Sucre et al., 2023; Rivas‐Chamorro et al., 2023; Xie et al., 2023). Different rates of evolution and different modes of inheritance could contribute to this incongruence. The results (Figures 4 and 6) implied that genetic drift process could play a minor role in generating discordant chloroplast and morphology phylogenies. This is because coalescent process by genetic drift is faster to reach the reciprocal monophyly for chloroplast genomes (1/*N*_e_, haploid) than for nuclear (1/2*N*_e_, diploid) genomes (Hu et al., 2019), which was not the case among the four varieties. Natural hybridization between varieties could occur in sympatry but likely had small effects on chloroplast genome divergence among varieties. This is because only seed dispersal contributes to gene flow of maternally inherited genes. A recent study indicated that the ratio of pollen to seed flow was substantial in the western region in *T. ciliata* complex (Xiao et al., 2023), which implies that effects of seed flow were much smaller those of pollen flow among sympatric varieties. A further clarification with nuclear genomes would aid in gaining insights into natural hybridization. This could also help to explain the incongruence between chloroplast genome‐and morphological trait‐based species delimitations.

An alternative explanation is that different models of selection likely participated in this conflict. The present results provided strong evidence that purifying selection acted on all protein‐coding genes except for the *ycf*2 gene, which could effectively impede chloroplast genome divergence among varieties. This likely differed from morphological traits (e.g., the leaf and flower traits) where adaptively divergent selection could be involved in and yielded extensive morphological diversity. Consequently, this produced discordant phylogenies in terms of morphological traits versus chloroplast genome sequences.

Only one protein‐coding gene, *ycf*2, was detected under weak positive selection. The *ycf*2 gene is the largest plastid gene in angiosperms (Drescher et al., 2000) and had 6822 bp in the four varieties. Previous studies indicated that the *ycf*2 gene had multiple positive selection sites during angiosperm evolution, and this gene was recommended for constructing angiosperm phylogeny due to its long sequence and a low rate of nucleotide substitution (Huang et al., 2010; Li, Ma, et al., 2019). The *ycf*2 gene was recently found to be essential for cell viability and a key enzyme for ATP production by chloroplast in the dark or in nonphotosynthetic plastids (Drescher et al., 2000; Kikuchi et al., 2018). Here, we found that *ycf*2 gene had two or four sites under positive selection from the tests on single gene sequence or on the concatenated sequence, respectively. Further investigation of this gene could be interested in relation to species delimitation.

Although a few amino acid sites were under positive selection, they accounted for only a small proportion of genome (Yang & Swanson, 2002). Most amino acid sites were under purifying selection. Thus, compared with the drift process (Freeland et al., 2011; Hudson & Coyne, 2002; Palumbi et al., 2001), purifying selection could be dominant in shaping chloroplast genome phylogeny at both gene and amino acid site levels, or in producing the conflict between morphological‐ and cpDNA‐based phylogenies.

### 4.3 Implications for genetic conservation

Previous studies according to the maximum entropy (MaxEnt) approach showed that *T. ciliata* var. *ciliata* would potentially expand as climate changes in the future, while *T. ciliata* var. *pubescens* would shrink in Yunnan Province (Zhang et al., 2018a, 2018b). The demographic changes in other two varieties remain unknown although the whole range of *T. ciliata* complex did not show expansion after bottleneck effects (Xiao et al., 2023). A strategy of conserving multiple populations was recommended in the western regions from the results assayed by nuclear ITS sequences.

This study identified four sympatric varieties of *T. ciliata* at the finer scale and further investigated genetic differentiation in sympatry in the western region. Our results support the strategy of conserving genetic variation based on the *T. ciliata* complex rather than on dividual variety. This is because a close genetic relationship existed among varieties of *T. ciliata*. Besides, natural hybridization among varieties could not be excluded in sympatry (Xiao et al., 2023). The traditional single variety‐based approach is not effective to conserve genetic variation of *T. ciliata* where hybrids between varieties are overlooked. Instead, the approach that maintains the evolutionary potential of the *T. ciliata* complex is more effective, such as the process‐based species conservation proposed by Ennos et al. (2012).

## 5 CONCLUSION

We investigated the phylogenetic relationship among four varieties of *T. ciliata* (*T. ciliata* var. *ciliata*, *T. ciliata* var. *yunnanensis*, *T. ciliata* var. *pubescens*, and *T. ciliata* var. *henryi*) in sympatry using the whole chloroplast genome sequences and two genetic markers. Comparative genomic analysis showed that genetic diversity among varieties was very small (*π* = 0.0003). These phylogenetic varieties were genetically well‐mixed, and their divergent times were about 0.92–24.47 Mya. Analysis with two markers indicated that a very small level of genetic differentiation occurred among varieties ($\Phi_{\mathrm{st}}$ = 0%–5%). Chloroplast genome‐based phylogeny was discordant with the morphology‐based species delimitation at the fine spatial scale (sympatric speciation). Strong purifying selection was detected across all protein‐coding genes except for the *ycf*2 gene that was under weak positive selection. A similar pattern of purifying selection was observed across genome‐wide amino acid sites. Purifying selection could play an important role in impeding chloroplast genome divergence among varieties. From these results, a conservation strategy focusing on the *T. ciliata* complex rather than individual variety is recommended for this endangered species in China.

## AUTHOR CONTRIBUTIONS

**Xin‐Sheng Hu:** Conceptualization (lead); funding acquisition (lead); project administration (lead); writing – review and editing (lead). **Yu Xiao:** Conceptualization (supporting); data curation (lead); formal analysis (lead); investigation (lead); methodology (lead); writing – original draft (lead). **Xi Wang:** Data curation (supporting); investigation (supporting); methodology (supporting); resources (supporting). **Zi‐Han He:** Data curation (supporting); investigation (supporting); resources (supporting). **Yan‐Wen Lv:** Data curation (supporting); investigation (supporting); resources (supporting). **Chun‐Hua Zhang:** Data curation (supporting); investigation (supporting); resources (supporting).

## Supporting information


Data S1.

## Data Availability

The complete sequences of chloroplast genomes of four varieties of *T. ciliata* were deposited in NCBI database. *Toona ciliata* var. *ciliata* (GenBank access numbers: OM135324, OP373439, and OP373440), *T. ciliata* var. *henryi* (OM135325, OP373441, and OP373442), *T. ciliata* var. *yunnanensis* (OM135326, OP373445, and OP373446), and *T. ciliata* var. *pubescens* (OM135327, OP373443, and OP373444). The *psa*A and *trn*L‐*trn*F alignment sequences were submitted to figshare (https://doi.org/10.6084/m9.figshare.24203763).
