# Supplementary material for: Assessing the phylogenetic relationship among varieties of Toona ciliata (Meliaceae) in sympatry with chloroplast genomes
Source: Ecol Evol. 2023 Dec 13;13(12):e10828. doi: 10.1002/ece3.10828 (PMC10716671; doi:10.1002/ece3.10828)

**Supplementary Information**

**Assessing the phylogenetic relationship among varieties of *Toona ciliata* (Meliaceae) in sympatry with chloroplast genomes**

Yu Xiao^1,2^, Xi Wang^1,2^, Zi-Han He^1,2^, Yan-Wen Lv^1,2^, Chun-Hua Zhang^3^, and Xin-Sheng Hu^1,2*^

^1^ College of Forestry and Landscape Architecture, South China Agricultural University, Guangzhou 510642, China

^2^ Guangdong Key Laboratory for Innovative Development and Utilization of Forest Plant Germplasm, Guangzhou 510642, China

^3^ Institute of Highland Forest Science, Chinese Academy of Forestry, Kunming 650233, China

^*^ Correspondence: xinsheng@scau.edu.cn

#### Table S1. Identification of four varieties of *T. ciliata* based on the leaf and flower traits

| Taxon | Flower | Leaf | Specimens |
| --- | --- | --- | --- |
| *T. ciliata* var. *ciliata* | Panicle terminal, as long as or slightly shorter than leaves, covered with short hard trichomes or subglabrous, pedicel short | Leaves even-pinnate or odd-pinnate, leaflets opposite to subopposite, blades oblong-ovate or lanceolate, both surfaces glabrous or abaxially appressed pubescent along veins | [KUN 0502111 (cvh.ac.cn)](https://www.cvh.ac.cn/spms/detail.php?id=0059f3fc)  (https://www.cvh.ac.cn/spms/detail.php?id=0059f3fc) |
| *T. ciliata* var. *yunnanensis* | Petals 5 × 3 mm, outside pubescent | Leaflets 5-10 × 3-5 cm, lowermost pair ovate, distal ovate-oblong to ovate-lanceolate | [KUN 1332939 (cvh.ac.cn)](https://www.cvh.ac.cn/spms/detail.php?id=1f986a80)  (https://www.cvh.ac.cn/spms/detail.php?id=1f986a80) |
| *T. ciliata* var. *pubescens* | Petals subovate-oblong, apex acuminate | Leaf rachis and leaflet abaxially pubescent, petiole ca. 9 mm | [PE 01168244 (cvh.ac.cn)](https://www.cvh.ac.cn/spms/detail.php?id=ee62fa60)  (https://www.cvh.ac.cn/spms/detail.php?id=ee62fa60) |
| *T. ciliata* var. *henryi* | Petals elliptic, rounded at both ends, filaments sparsely pilose, panicle shorter than leaves | Leaflets 12 × 4.5 cm, adaxially puberulent along midvein veins, abaxially glabrous or sparsely puberulent along vein, base attenuate | [IMDY IMDY0013495 (cvh.ac.cn)](https://www.cvh.ac.cn/spms/detail.php?id=ef61156d)  (https://www.cvh.ac.cn/spms/detail.php?id=ef61156d) |
| *T. ciliata* var. *sublaxiflora* | Petals oblong, apex acuminate, filaments sparsely pilose | Leaflets alternate or opposite, 13 × 3.5 cm, blades oblong-lanceolate, apex acuminate |  |

**Table S2**. Sample information and geographic coordinates of four varieties of *T. ciliata*. Twelve individuals for whole genome sequencing were marked in bold

| Sample | Location | Latitutde  (N) | Longitude  (E) | Altitude  (m) |
| --- | --- | --- | --- | --- |
| *T. ciliata* var. ciliata 1 | **Lijiang, Yunnan** | **26°24′19′′** | **100°39′17′′** | **1482.15** |
| *T. ciliata* var. ciliata 2 | Puer, Yunnan | 24°42′16′′ | 100°39′25′′ | 1295.94 |
| *T. ciliata* var. ciliata 3 | Puer, Yunnan | 24°42′16′′ | 100°39′28′′ | 1309.40 |
| *T. ciliata* var. ciliata 4 | Puer, Yunnan | 24°39′56′′ | 100°42′24′′ | 1265.88 |
| *T. ciliata* var. ciliata 5 | **Puer, Yunnan** | **24°32′29′′** | **100°46′56′′** | **1181.68** |
| *T. ciliata* var. ciliata 6 | Puer, Yunnan | 24°20′51′′ | 100°54′60′′ | 1092.28 |
| *T. ciliata* var. ciliata 7 | Puer, Yunnan | 24°15′08′′ | 100°57′27′′ | 1108.38 |
| *T. ciliata* var. ciliata 8 | Puer, Yunnan | 24°23′27′′ | 100°52′53′′ | 1154.72 |
| *T. ciliata* var. ciliata 9 | Puer, Yunnan | 24°21′37′′ | 100°51′43′′ | 1411.40 |
| *T. ciliata* var. ciliata 10 | Puer, Yunnan | 24°24′40′′ | 100°52′56′′ | 1145.07 |
| *T. ciliata* var. ciliata 11 | Puer, Yunnan | 24°25′37′′ | 100°52′20′′ | 1112.69 |
| *T. ciliata* var. ciliata 12 | **Puer, Yunnan** | **24°25′39′′** | **100°52′16′′** | **1116.94** |
| *T. ciliata* var. pubescens 1 | Kunming, Yunnan | 25°38′39′′ | 102°38′54′′ | 1434.49 |
| *T. ciliata* var. pubescens 2 | Kunming, Yunnan | 25°38′40′′ | 102°38′53′′ | 1444.59 |
| *T. ciliata* var. pubescens 3 | Kunming, Yunnan | 25°38′53′′ | 102°38′49′′ | 1427.22 |
| *T. ciliata* var. pubescens 4 | Kunming, Yunnan | 25°43′01′′ | 102°39′16′′ | 1476.38 |
| *T. ciliata* var. pubescens 5 | **Kunming, Yunnan** | **25°43′10′′** | **102°39′34′′** | **1448.33** |
| *T. ciliata* var. pubescens 6 | Kunming, Yunnan | 25°43′38′′ | 102°39′29′′ | 1392.63 |
| *T. ciliata* var. pubescens 7 | Kunming, Yunnan | 25°43′43′′ | 102°39′29′′ | 1370.78 |
| *T. ciliata* var. pubescens 8 | Kunming, Yunnan | 25°43′48′′ | 102°39′40′′ | 1352.01 |
| *T. ciliata* var. pubescens 9 | Kunming, Yunnan | 25°43′54′′ | 102°39′50′′ | 1341.40 |
| *T. ciliata* var. pubescens 10 | Kunming, Yunnan | 25°38′45′′ | 102°38′49′′ | 1504.78 |
| *T. ciliata* var. pubescens 11 | Kunming, Yunnan | 25°38′46′′ | 102°38′48′′ | 1501.62 |
| *T. ciliata* var. pubescens 12 | Kunming, Yunnan | 25°39′13′′ | 102°38′12′′ | 1540.96 |
| *T. ciliata* var. pubescens 13 | Lijiang, Yunnan | 26°33′54′′ | 101°22′24′′ | 1047.75 |
| *T. ciliata* var. pubescens 14 | Lijiang, Yunnan | 26°34′29′′ | 101°18′40′′ | 1173.31 |
| *T. ciliata* var. pubescens 15 | Lijiang, Yunnan | 26°31′16′′ | 101°14′43′′ | 1222.58 |
| *T. ciliata* var. pubescens 16 | **Lijiang, Yunnan** | **26°29′16′′** | **101°12′30′′** | **1571.29** |
| *T. ciliata* var. pubescens 17 | Lijiang, Yunnan | 26°27′37′′ | 101°09′13′′ | 1987.49 |
| *T. ciliata* var. pubescens 18 | Lijiang, Yunnan | 26°27′54′′ | 101°00′45′′ | 1372.71 |
| *T. ciliata* var. pubescens 19 | Lijiang, Yunnan | 26°30′20′′ | 100°59′18′′ | 1361.80 |
| *T. ciliata* var. pubescens 20 | **Lijiang, Yunnan** | **26°24′13′′** | **100°39′14′′** | **1487.95** |
| *T. ciliata* var. yunnanensis 1 | **Kunming, Yunnan** | **25°38′43′′** | **102°38′54′′** | **1438.68** |
| *T. ciliata* var. yunnanensis 2 | Kunming, Yunnan | 25°39′04′′ | 102°38′45′′ | 1419.92 |
| *T. ciliata* var. yunnanensis 3 | Lijiang, Yunnan | 26°33′54′′ | 101°22′24′′ | 1047.75 |
| *T. ciliata* var. yunnanensis 4 | **Lijiang, Yunnan** | **26°34′30′′** | **101°18′41′′** | **1175.75** |
| *T. ciliata* var. yunnanensis 5 | Lijiang, Yunnan | 26°24′13′′ | 100°39′15′′ | 1490.97 |
| *T. ciliata* var. yunnanensis 6 | Lijiang, Yunnan | 26°24′15′′ | 100°39′15′′ | 1499.01 |
| *T. ciliata* var. yunnanensis 7 | Lijiang, Yunnan | 26°24′15′′ | 100°39′16′′ | 1495.61 |
| *T. ciliata* var. yunnanensis 8 | **Lijiang, Yunnan** | **26°24′17′′** | **100°39′16′′** | **1497.12** |
| *T. ciliata* var. yunnanensis 9 | Lijiang, Yunnan | 26°24′18′′ | 100°39′17′′ | 1492.85 |
| *T. ciliata* var. yunnanensis 10 | Lijiang, Yunnan | 26°24′19′′ | 100°39′17′′ | 1488.64 |
| *T. ciliata* var. yunnanensis 11 | Lijiang, Yunnan | 26°23′00′′ | 100°38′26′′ | 1470.75 |
| *T. ciliata* var. yunnanensis 12 | Lijiang, Yunnan | 26°22′52′′ | 100°38′20′′ | 1466.59 |
| *T. ciliata* var. yunnanensis 13 | Lijiang, Yunnan | 26°22′24′′ | 100°37′59′′ | 1438.94 |
| *T. ciliata* var. henryi 1 | **Puer, Yunnan** | **24°32′60′′** | **100°47′39′′** | **1250.22** |
| *T. ciliata* var. henryi 2 | Puer, Yunnan | 24°32′10′′ | 100°47′37′′ | 1254.11 |
| *T. ciliata* var. henryi 3 | Puer, Yunnan | 24°31′55′′ | 100°47′39′′ | 1281.97 |
| *T. ciliata* var. henryi 4 | Puer, Yunnan | 24°2920 | 100°4710 | 1296.19 |
| *T. ciliata* var. henryi 5 | Puer, Yunnan | 24°2920 | 100°4710 | 1296.56 |
| *T. ciliata* var. henryi 6 | Puer, Yunnan | 24°2920 | 100°4710 | 1295.89 |
| *T. ciliata* var. henryi 7 | Puer, Yunnan | 24°1847 | 100°5534 | 1092.56 |
| *T. ciliata* var. henryi 8 | **Puer, Yunnan** | **24°1134** | **100°5838** | **1085.08** |
| *T. ciliata* var. henryi 9 | Puer, Yunnan | 24°1031 | 100°5840 | 1094.01 |
| *T. ciliata* var. henryi 10 | Puer, Yunnan | 24°1021 | 100°5837 | 1078.82 |
| *T. ciliata* var. henryi 11 | Puer, Yunnan | 24°1019 | 100°5837 | 1081.71 |
| *T. ciliata* var. henryi 12 | Puer, Yunnan | 24°0738 | 101°0231 | 1079.98 |
| *T. ciliata* var. henryi 13 | **Puer, Yunnan** | **24°0056** | **101°0301** | **1070.95** |

**Table S3**. Chloroplast genome sequencing and assembly information for three individuals of *T. ciliata* var. *ciliata*, *T. ciliata* var. *pubescens*, *T. ciliata* var. *henryi* and *T. ciliata* var. *yunnanensis*

| Sample | Clean reads (bp) | Clean base  (bp) | Assembly reads  (bp) | Assembly bases  (bp) | Q20  (%) | Q30  (%) | Average sequencing depth (X) | Coverage rate  (%) |
| --- | --- | --- | --- | --- | --- | --- | --- | --- |
| *T. ciliata* var. ciliata 1 | 15,183,304 | 2,277,495,600 | 292,224 | 43,833,600 | 97.92 | 93.79 | 252.94 | 100 |
| *T. ciliata* var. ciliata 5 | 24,955,476 | 3,743,321,400 | 630,900 | 94,635,000 | 96.58 | 91.09 | 539.34 | 100 |
| *T. ciliata* var. ciliata 12 | 29,012,334 | 4,351,850,100 | 874,506 | 131,175,900 | 96.14 | 90.19 | 720.79 | 100 |
| *T. ciliata* var. pubescens 5 | 16,961,802 | 2,544,270,300 | 497,372 | 74,605,800 | 96.76 | 91.39 | 419.26 | 100 |
| *T. ciliata* var. pubescens 16 | 27,005,674 | 4,050,851,100 | 298,248 | 44,737,200 | 97.87 | 93.78 | 245.48 | 100 |
| *T. ciliata* var. pubescens 20 | 21,096,392 | 3,164,458,800 | 234,892 | 35,233,800 | 96.41 | 90.70 | 186.69 | 100 |
| *T. ciliata* var. yunnanensis 1 | 15,869,354 | 2,380,403,100 | 211,834 | 31,775,100 | 97.75 | 93.39 | 179.61 | 100 |
| *T. ciliata* var. yunnanensis 4 | 24,758,864 | 3,713,829,600 | 425,278 | 63,791,700 | 96.56 | 90.83 | 341.48 | 100 |
| *T. ciliata* var. yunnanensis 8 | 21,295,618 | 3,194,342,700 | 1,492,148 | 223,822,200 | 96.97 | 91.93 | 1101.46 | 100 |
| *T. ciliata* var. henryi 1 | 15,034,170 | 2,255,125,500 | 315,040 | 47,256,000 | 97.78 | 93.64 | 265.04 | 100 |
| *T. ciliata* var. henryi 8 | 14,126.602 | 2,118,990,300 | 384,504 | 57,675,600 | 96.60 | 91.23 | 312.83 | 100 |
| *T. ciliata* var. henryi 13 | 28,583,042 | 4,287,456,300 | 675,988 | 101,398,200 | 95.96 | 89.80 | 582.58 | 100 |

#### Table S4. Distribution of repetitive sequences in chloroplast genomes of *T. ciliata* var. *ciliata*, *T. ciliata* var. *henryi* and *T. ciliata* var. *yunnanensis*

| No. | Size (bp) | Type | Number of repeats | Location | Region |
| --- | --- | --- | --- | --- | --- |
| 1 | 30 | Forward | 1 | IGS(tRNA-Ser(GCT), tRNA-Gly(TCC))  IGS(*psb*M, tRNA-Asp(GTC)) | LSC |
| 2 | 30 | Palindromic | 1 | IGS(tRNA-Ser(GCT), tRNA-Gly(TCC))  IGS(*rps*7, *ycf*15) | LSC, IRA |
| 3 | 30 | Forward | 1 | IGS(tRNA-Ser(GCT), tRNA-Gly(TCC))  IGS(*ycf*15, *rps*12) | LSC, IRB |
| 4 | 30 | Palindromic | 1 | intron_*pet*D | LSC |
| 5 | 30 | Forward | 1 | *ycf*2 | IRA |
| 6 | 30 | Palindromic | 2 | *ycf*2 | IRA, IRB |
| 7 | 31 | Palindromic | 1 | intron_*ycf*3 | LSC |
| 8 | 31 | Forward | 1 | intron_*clp*P  intron_*ndh*B | LSC, IRA |
| 9 | 31 | Palindromic | 1 | intron_*clp*P  intron_*ndh*B | LSC, IRB |
| 10 | 31 | Complement | 1 | IGS(*rps*7, *ycf*15)  IGS(*ycf*15, *rps*12) | IRA, IRB |
| 11 | 34 | Palindromic | 1 | intron_*rps*16 | LSC |
| 12 | 34 | Palindromic | 1 | IGS(*rps*4, tRNA-Thr(TGT)) | LSC |
| 13 | 34 | Forward | 1 | IGS(rrn4.5S, rrn5S)  IGS(tRNA-Asn(GTT), *ndh*F) | IRA |
| 14 | 34 | Palindromic | 2 | IGS(rrn4.5S, rrn5S)  IGS(rrn5S, rrn4.5S) | IRA, IRB |
| 15 | 34 | Forward | 1 | IGS(rrn5S, rrn4.5S) | IRB |
| 16 | 35 | Palindromic | 1 | IGS(tRNA-Thr(GGT), *psb*D) | LSC |
| 17 | 35 | Palindromic | 1 | intron_*ycf*3  intron_*pet*B | LSC |
| 18 | 36 | Forward | 1 | intron_*ycf*3  intron_*ndh*A | LSC, SSC |
| 19 | 39 | Forward | 1 | intron_*ycf*3  IGS(*rps*7, *ycf*15) | LSC, IRA |
| 20 | 39 | Palindromic | 1 | intron_*ycf*3  IGS(*ycf*15, *rps*12) | LSC, IRB |
| 21 | 41 | Forward | 1 | *psa*B  *psa*A | LSC |
| 22 | 41 | Forward | 1 | IGS(*rps*7, *ycf*15)  *ndh*F | IRA, SSC |
| 23 | 41 | Palindromic | 1 | intron_*ndh*A  IGS(*ycf*15, *rps*12) | SSC, IRB |
| 24 | 43 | Reverse | 1 | intron_*clp*P | LSC |
| 25 | 44 | Palindromic | 1 | IGS(*psb*T, *psb*N) | LSC |
| 26 | 46 | Forward | 1 | *ycf*2 | IRA |
| 27 | 46 | Palindromic | 2 | *ycf*2 | IRA, IRB |
| 28 | 46 | Forward | 1 | *ycf*2 | IRB |
| 29 | 48 | Palindromic | 1 | IGS(*pet*N, *psb*M) | LSC |
| 30 | 51 | Palindromic | 1 | IGS(tRNA-Gly(TCC), tRNA-Arg(TCT)) | LSC |
| 31 | 58 | Palindromic | 1 | IGS(tRNA-His(GTG), *psb*A) | LSC |

#### Table S5. Distribution of repetitive sequences in chloroplast genome of *T. ciliata* var. *pubescens*

| No. | Size (bp) | Type | Number of repeat | Location | Region |
| --- | --- | --- | --- | --- | --- |
| 1 | 30 | Palindromic | 1 | intron_*pet*D | LSC |
| 2 | 30 | Forward | 1 | *ycf*2 | IRA |
| 3 | 30 | Palindromic | 2 | *ycf*2 | IRA, IRB |
| 4 | 31 | Forward | 1 | IGS(tRNA-Ser(GCT), tRNA-Gly(TCC))  IGS(*psb*M, tRNA-Asp(GTC)) | LSC |
| 5 | 31 | Palindromic | 1 | intron_*ycf*3 | LSC |
| 6 | 31 | Complement | 1 | IGS(*rps*7, *ycf*15)  IGS(*ycf*15, *rps*12) | IRA, IRB |
| 7 | 32 | Palindromic | 1 | intron_*rps*16 | LSC |
| 8 | 32 | Forward | 1 | intron_*clp*P  intron_*ndh*B | LSC, IRA |
| 9 | 32 | Palindromic | 1 | intron_*clp*P  intron_*ndh*B | LSC, IRB |
| 10 | 34 | Palindromic | 1 | IGS(*rps*4, tRNA-Thr(TGT)) | LSC |
| 11 | 34 | Forward | 1 | IGS(rrn4.5S, rrn5S)  IGS(tRNA-Asn(GTT), *ndh*F) | IRA |
| 12 | 34 | Palindromic | 2 | IGS(rrn4.5S, rrn5S)  IGS(rrn5S, rrn4.5S) | IRA. IRB |
| 13 | 34 | Forward | 1 | IGS(rrn5S, rrn4.5S) | IRB |
| 14 | 35 | Palindromic | 1 | IGS(tRNA-Thr(GGT), *psb*D) | LSC |
| 15 | 35 | Palindromic | 1 | intron_*ycf*3  intron_*pet*B | LSC |
| 16 | 36 | Forward | 1 | intron_*ycf*3  intron_*ndh*A | LSC. SSC |
| 17 | 39 | Forward | 1 | intron_*ycf*3  IGS(*rps*7, *ycf*15) | LSC, IRA |
| 18 | 39 | Palindromic | 1 | intron_*ycf*3  IGS(*ycf*15, *rps*12) | LSC, IRB |
| 19 | 41 | Forward | 1 | *psa*B  *psa*A | LSC |
| 20 | 41 | Forward | 1 | IGS(*rps*7, *ycf*15)  *ndh*F | IRA, SSC |
| 21 | 41 | Palindromic | 1 | intron_*ndh*A  IGS(*ycf*15, *rps*12) | SSC, IRB |
| 22 | 44 | Palindromic | 1 | IGS(*psb*T, *psb*N) | LSC |
| 23 | 46 | Forward | 1 | *ycf*2 | IRA |
| 24 | 46 | Palindromic | 2 | *ycf*2 | IRA, IRB |
| 25 | 46 | Forward | 1 | *ycf*2 | IRB |
| 26 | 48 | Palindromic | 1 | IGS(*pet*N, *psb*M) | LSC |
| 27 | 51 | Palindromic | 1 | IGS(tRNA-Gly(TCC),tRNA-Arg(TCT)) | LSC |
| 28 | 58 | Palindromic | 1 | IGS(tRNA-His(GTG), *psb*A) | LSC |

#### Table S6. Parameter estimates and log-likelihood tests under one-and two-$\boldsymbol{\omega}$ ratios branch models

| Gene | one-$\omega$ ratio model | | two-$\omega$ ratio models | | | | | | | | | | | |
| --- | --- | --- | --- | --- | --- | --- | --- | --- | --- | --- | --- | --- | --- | --- |
|  | lnL | Estimates of parameters | *T. ciliata* var. *ciliata* | | | *T. ciliata* var. *henryi* | | | *T. ciliata* var. *yunnanensis* | | | *T. ciliata* var. *pubescens* | | |
|  |  |  | lnL | 2$\Delta$LRT | *p*-value | lnL | $2\Delta$LRT | *p*-value | lnL | $2\Delta$LRT | *p*-value | lnL | $2\Delta$LRT | *p*-value |
|  |  | $\omega_{0}$ |  |  |  |  |  |  |  |  |  |  |  |  |
| *rps*12 | -509.5431 | 0.0001 | -509.5431 | 0.00007 | 0.99 | -509.5431 | 0.00006 | 0.99 | -509.5431 | 0.00006 | 0.99 | -509.5432 | 0.00018 | 0.99 |
| *psb*A | -1518.8145 | 0.0001 | -1518.8146 | 0.00009 | 0.99 | -1518.8146 | 0.00010 | 0.99 | -1518.8148 | 0.00056 | 0.98 | -1518.8145 | 0.00007 | 0.99 |
| *mat*K | -2448.0554 | 0.5898 | -2448.0554 | 0.00001 | 1.00 | -2448.0554 | 0.00001 | 1.00 | -2446.9686 | 2.17354 | 0.14 | -2447.6350 | 0.84068 | 0.36 |
| *rps*16 | -363.4625 | 0.3717 | -363.4624 | 0.00000 | 1.00 | -363.4624 | 0.00000 | 1.00 | -363.4624 | 0.00001 | 1.00 | -363.4624 | 0.00000 | 1.00 |
| *psb*K | -254.1235 | 0.2239 | -254.1233 | 0.00035 | 0.98 | -254.1233 | 0.00035 | 0.98 | -254.1233 | 0.00035 | 0.98 | -254.1233 | 0.00035 | 0.98 |
| *psb*I | -138.3714 | 1.5000 | -138.3714 | 0.00000 | 1.00 | -138.3714 | 0.00000 | 1.00 | -138.3714 | 0.00001 | 1.00 | -138.3714 | 0.00000 | 1.00 |
| *atp*A | -2155.8746 | 0.0275 | -2155.8746 | 0.00002 | 1.00 | -2155.8747 | 0.00016 | 0.99 | -2155.8747 | 0.00016 | 0.99 | -2155.8747 | 0.00014 | 0.99 |
| *atp*F | -835.1782 | 0.4531 | -835.1783 | 0.00022 | 0.99 | -835.1781 | 0.00013 | 0.99 | -835.1782 | 0.00018 | 0.99 | -835.1781 | 0.00013 | 0.99 |
| *atp*H | -318.4154 | 0.0001 | -318.4154 | 0.00002 | 1.00 | -318.4154 | 0.00002 | 1.00 | -318.4154 | 0.00002 | 1.00 | -318.4155 | 0.00011 | 0.99 |
| *atp*I | -1072.0860 | 0.0640 | -1072.0861 | 0.00014 | 0.99 | -1072.0861 | 0.00023 | 0.99 | -1072.0860 | 0.00004 | 1.00 | -1072.0860 | 0.00004 | 1.00 |
| *rps*2 | -1031.0581 | 0.1358 | -1030.9572 | 0.20178 | 0.65 | -1030.9572 | 0.20178 | 0.65 | -1030.9572 | 0.20185 | 0.65 | -1030.9572 | 0.20178 | 0.65 |
| *rpo*C2 | -6330.8221 | 0.2045 | -6330.8222 | 0.00013 | 0.99 | -6330.8222 | 0.00014 | 0.99 | -6330.8222 | 0.00013 | 0.99 | -6330.8073 | 0.02965 | 0.86 |
| *rpo*C1 | -2905.5348 | 0.1602 | -2905.5348 | 0.00012 | 0.99 | -2905.5348 | 0.00012 | 0.99 | -2905.5348 | 0.00011 | 0.99 | -2904.5460 | 1.97755 | 0.16 |
| *rpo*B | -4681.7218 | 0.1111 | -4681.7219 | 0.00014 | 0.99 | -4681.7219 | 0.00013 | 0.99 | -4681.7219 | 0.00014 | 0.99 | -4681.3912 | 0.66136 | 0.42 |
| *pet*N | -108.7142 | 1.5000 | -108.7142 | 0.00002 | 1.00 | -108.7142 | 0.00001 | 1.00 | -108.7142 | 0.00000 | 1.00 | -108.7142 | 0.00001 | 1.00 |
| *psb*M | -128.2382 | 0.0001 | -128.2382 | 0.00002 | 1.00 | -128.2382 | 0.00002 | 1.00 | -128.2388 | 0.00117 | 0.97 | -128.2382 | 0.00002 | 1.00 |
| *psb*D | -1493.1498 | 0.0001 | -1493.1486 | 0.00242 | 0.96 | -1493.1486 | 0.00241 | 0.96 | -1493.1495 | 0.00060 | 0.98 | -1493.1486 | 0.00241 | 0.96 |
| *psb*C | -2021.9980 | 0.0315 | -2021.9981 | 0.00016 | 0.99 | -2021.9980 | 0.00002 | 1.00 | -2021.9981 | 0.00016 | 0.99 | -2021.9078 | 0.18059 | 0.67 |
| *psb*Z | -266.8188 | 0.0563 | -266.8188 | 0.00001 | 1.00 | -266.8188 | 0.00001 | 1.00 | -266.8188 | 0.00001 | 1.00 | -266.8188 | 0.00000 | 1.00 |
| *rps*14 | -418.5082 | 0.0001 | -418.5082 | 0.00000 | 1.00 | -418.5082 | 0.00000 | 1.00 | -418.5082 | 0.00000 | 1.00 | -418.5082 | 0.00000 | 1.00 |
| *psa*B | -3047.4266 | 0.0001 | -3047.4267 | 0.00007 | 0.99 | -3047.4267 | 0.00007 | 0.99 | -3047.4269 | 0.00058 | 0.98 | -3047.4270 | 0.00082 | 0.98 |
| *psa*A | -3270.1209 | 0.0423 | -3271.3299 | 2.41795 | 0.12 | -3271.3297 | 2.41754 | 0.12 | -3268.5772 | 3.08730 | 0.08 | -3269.3537 | 1.53445 | 0.22 |
| *ycf*3 | -704.4286 | 0.0557 | -704.4286 | 0.00001 | 1.00 | -704.4286 | 0.00001 | 1.00 | -704.4286 | 0.00001 | 1.00 | -704.4286 | 0.00001 | 1.00 |
| *rps*4 | -828.8875 | 0.0393 | -828.8876 | 0.00006 | 0.99 | -828.8876 | 0.00006 | 0.99 | -828.8876 | 0.00006 | 0.99 | -828.8876 | 0.00005 | 0.99 |
| *ndh*J | -669.6517 | 0.0001 | -669.6517 | 0.00000 | 1.00 | -669.6517 | 0.00000 | 1.00 | -669.6517 | 0.00000 | 1.00 | -669.6518 | 0.00009 | 0.99 |
| *ndh*K | -1011.3749 | 0.1246 | -1011.3749 | 0.00003 | 1.00 | -1011.3749 | 0.00003 | 1.00 | -1011.3749 | 0.00003 | 1.00 | -1011.3749 | 0.00003 | 1.00 |
| *ndh*C | -492.6415 | 0.1080 | -492.6415 | 0.00003 | 1.00 | -492.6415 | 0.00003 | 1.00 | -492.6415 | 0.00012 | 0.99 | -492.6415 | 0.00012 | 0.99 |
| *atp*E | -586.8742 | 0.1597 | -586.8742 | 0.00001 | 1.00 | -586.8742 | 0.00001 | 1.00 | -586.8742 | 0.00001 | 1.00 | -586.4312 | 0.88589 | 0.35 |
| *atp*B | -2180.3254 | 0.0458 | -2180.3254 | 0.00009 | 0.99 | -2180.3254 | 0.00009 | 0.99 | -2180.3254 | 0.00009 | 0.99 | -2180.1987 | 0.25336 | 0.61 |
| *rbc*L | -2082.6955 | 0.0816 | -2082.6956 | 0.00015 | 0.99 | -2082.6959 | 0.00076 | 0.98 | -2082.6954 | 0.00031 | 0.99 | -2082.6951 | 0.00087 | 0.98 |
| *acc*D | -2227.4802 | 0.3496 | -2227.4802 | 0.00014 | 0.99 | -2227.4802 | 0.00014 | 0.99 | -2227.4803 | 0.00007 | 0.99 | -2227.4803 | 0.00006 | 0.99 |
| *psa*I | -156.3308 | 0.2442 | -156.3308 | 0.00003 | 1.00 | -156.3308 | 0.00003 | 1.00 | -156.3308 | 0.00004 | 1.00 | -156.3308 | 0.00003 | 1.00 |
| *ycf*4 | -815.6092 | 0.5141 | -815.6092 | 0.00002 | 1.00 | -815.6092 | 0.00002 | 1.00 | -815.6092 | 0.00001 | 1.00 | -815.6091 | 0.00005 | 0.99 |
| *cem*A | -1060.7791 | 0.4838 | -1060.7791 | 0.00000 | 1.00 | -1060.7791 | 0.00000 | 1.00 | -1060.7791 | 0.00000 | 1.00 | -1059.8929 | 1.77249 | 0.18 |
| *pet*A | -1433.2105 | 0.2822 | -1433.2105 | 0.00005 | 0.99 | -1433.2105 | 0.00005 | 0.99 | -1433.2105 | 0.00005 | 0.99 | -1432.4852 | 1.45064 | 0.23 |
| *psb*J | -151.0831 | 1.5000 | -151.0831 | 0.00000 | 1.00 | -151.0831 | 0.00000 | 1.00 | -151.0831 | 0.00000 | 1.00 | -151.0830 | 0.00000 | 1.00 |
| *psb*L | -152.5536 | 0.0001 | -152.5538 | 0.00036 | 0.98 | -152.5536 | 0.00001 | 1.00 | -152.5536 | 0.00001 | 1.00 | -152.5536 | 0.00000 | 1.00 |
| *psb*F | -174.4815 | 0.0827 | -174.4816 | 0.00005 | 0.99 | -174.4816 | 0.00005 | 0.99 | -174.4816 | 0.00005 | 0.99 | -174.4816 | 0.00005 | 0.99 |
| *psb*E | -349.4187 | 0.1313 | -349.4187 | 0.00002 | 1.00 | -349.4187 | 0.00002 | 1.00 | -349.4187 | 0.00002 | 1.00 | -349.4187 | 0.00002 | 1.00 |
| *pet*L | -114.8258 | 0.0001 | -114.8258 | 0.00005 | 0.99 | -114.8258 | 0.00005 | 0.99 | -114.8258 | 0.00005 | 0.99 | -114.8258 | 0.00005 | 0.99 |
| *pet*G | -149.1094 | 0.0001 | -149.1094 | 0.00000 | 1.00 | -149.1094 | 0.00000 | 1.00 | -149.1094 | 0.00000 | 1.00 | -149.1094 | 0.00000 | 1.00 |
| *psa*J | -174.5635 | 0.3716 | -174.5634 | 0.00029 | 0.99 | -174.5634 | 0.00029 | 0.99 | -174.5634 | 0.00029 | 0.99 | -174.5635 | 0.00001 | 1.00 |
| *rpl*33 | -281.2596 | 0.4731 | -281.2596 | 0.00001 | 1.00 | -281.2596 | 0.00001 | 1.00 | -281.2596 | 0.00001 | 1.00 | -281.2596 | 0.00001 | 1.00 |
| *rps*18 | -432.6244 | 0.3336 | -432.6244 | 0.00002 | 1.00 | -432.6244 | 0.00002 | 1.00 | -432.6244 | 0.00002 | 1.00 | -432.6244 | 0.00002 | 1.00 |
| *rpl*20 | -518.7471 | 0.2532 | -518.7471 | 0.00001 | 1.00 | -518.7471 | 0.00001 | 1.00 | -517.9697 | 1.55478 | 0.21 | -518.7471 | 0.00001 | 1.00 |
| *clp*P | -857.3255 | 0.2527 | -857.3255 | 0.00006 | 0.99 | -857.3255 | 0.00006 | 0.99 | -857.3255 | 0.00006 | 0.99 | -856.8077 | 1.03562 | 0.31 |
| *psb*B | -2141.5331 | 0.0382 | -2141.5332 | 0.00017 | 0.99 | -2141.5332 | 0.00017 | 0.99 | -2141.5332 | 0.00017 | 0.99 | -2141.4227 | 0.22070 | 0.64 |
| *psb*T | -134.3386 | 0.0001 | -134.3386 | 0.00002 | 1.00 | -134.3386 | 0.00002 | 1.00 | -134.3386 | 0.00000 | 1.00 | -134.3386 | 0.00000 | 1.00 |
| *psb*N | -190.7499 | 0.0001 | -190.7499 | 0.00000 | 1.00 | -190.7499 | 0.00000 | 1.00 | -190.7499 | 0.00000 | 1.00 | -190.7499 | 0.00000 | 1.00 |
| *psb*H | -338.3976 | 0.1295 | -338.3976 | 0.00002 | 1.00 | -338.3976 | 0.00002 | 1.00 | -338.3976 | 0.00001 | 1.00 | -338.3976 | 0.00001 | 1.00 |
| *pet*B | -911.0382 | 0.0687 | -911.0383 | 0.00010 | 0.99 | -911.0383 | 0.00010 | 0.99 | -911.0383 | 0.00009 | 0.99 | -911.0383 | 0.00009 | 0.99 |
| *pet*D | -694.9773 | 0.0565 | -694.9772 | 0.00033 | 0.99 | -694.9771 | 0.00051 | 0.98 | -694.9774 | 0.00006 | 0.99 | -694.9773 | 0.00013 | 0.99 |
| *rpo*A | -1453.0279 | 0.1287 | -1453.0279 | 0.00004 | 0.99 | -1453.0279 | 0.00004 | 0.99 | -1453.0279 | 0.00004 | 0.99 | -1453.0279 | 0.00004 | 0.99 |
| *rps*11 | -594.9977 | 0.0511 | -594.9977 | 0.00001 | 1.00 | -594.9977 | 0.00005 | 0.99 | -594.9977 | 0.00006 | 0.99 | -594.9977 | 0.00006 | 0.99 |
| *rpl*36 | -169.4014 | 0.0563 | -169.4014 | 0.00001 | 1.00 | -169.4014 | 0.00001 | 1.00 | -169.4014 | 0.00001 | 1.00 | -169.4014 | 0.00001 | 1.00 |
| *rps*8 | -652.3814 | 0.4126 | -652.3814 | 0.00002 | 1.00 | -652.3814 | 0.00002 | 1.00 | -652.3814 | 0.00002 | 1.00 | -652.3814 | 0.00002 | 1.00 |
| *rpl*14 | -514.8886 | 0.0414 | -514.8886 | 0.00001 | 1.00 | -514.8886 | 0.00001 | 1.00 | -514.8886 | 0.00001 | 1.00 | -514.8886 | 0.00001 | 1.00 |
| *rpl*16 | -562.8476 | 0.0417 | -562.8479 | 0.00060 | 0.98 | -562.8476 | 0.00003 | 1.00 | -562.8478 | 0.00043 | 0.98 | -562.8476 | 0.00004 | 1.00 |
| *rps*3 | -928.5413 | 0.1009 | -928.5413 | 0.00004 | 1.00 | -928.5413 | 0.00004 | 1.00 | -928.5413 | 0.00003 | 1.00 | -928.5413 | 0.00002 | 1.00 |
| *rpl*22 | -716.8796 | 0.3280 | -716.8796 | 0.00002 | 1.00 | -716.8796 | 0.00001 | 1.00 | -716.2722 | 1.21486 | 0.27 | -716.8796 | 0.00001 | 1.00 |
| *rps*19 | -371.7989 | 0.1354 | -371.7988 | 0.00008 | 0.99 | -371.7988 | 0.00008 | 0.99 | -371.7988 | 0.00008 | 0.99 | -371.7991 | 0.00057 | 0.98 |
| *rpl*2 | -1096.5881 | 0.0001 | -1096.5881 | 0.00007 | 0.99 | -1096.5881 | 0.00007 | 0.99 | -1096.5881 | 0.00007 | 0.99 | -1096.5881 | 0.00007 | 0.99 |
| *rpl*23 | -378.7187 | 999.0000 | -378.7186 | 0.00009 | 0.99 | -378.7186 | 0.00009 | 0.99 | -378.7186 | 0.00009 | 0.99 | -378.7186 | 0.00009 | 0.99 |
| *ycf*2 | -9651.3072 | 1.0699 | -9651.3060 | 0.00237 | 0.96 | -9651.3057 | 0.00295 | 0.96 | -9651.5778 | 0.54127 | 0.46 | -9651.3060 | 0.00233 | 0.96 |
| *ndh*B | -2082.1563 | 0.1726 | -2082.1564 | 0.00010 | 0.99 | -2082.1564 | 0.00011 | 0.99 | -2082.1564 | 0.00010 | 0.99 | -2082.1564 | 0.00010 | 0.99 |
| *rps*7 | -608.1924 | 1.5000 | -608.1923 | 0.00032 | 0.99 | -608.1924 | 0.00001 | 1.00 | -608.1924 | 0.00000 | 1.00 | -608.1924 | 0.00000 | 1.00 |
| *ycf*15 | -252.8644 | 0.0001 | -252.8640 | 0.00077 | 0.98 | -252.8640 | 0.00077 | 0.98 | -252.8640 | 0.00077 | 0.98 | -252.8640 | 0.00077 | 0.98 |
| *ndh*F | -3459.7674 | 0.2247 | -3459.7675 | 0.00011 | 0.99 | -3459.7675 | 0.00013 | 0.99 | -3459.7675 | 0.00012 | 0.99 | -3459.7675 | 0.00014 | 0.99 |
| *rpl*32 | -268.3238 | 0.2990 | -268.3238 | 0.00000 | 1.00 | -268.3238 | 0.00000 | 1.00 | -268.3238 | 0.00000 | 1.00 | -268.3238 | 0.00000 | 1.00 |
| *ccs*A | -1447.3026 | 0.1395 | -1447.2359 | 0.13333 | 0.71 | -1447.2359 | 0.13333 | 0.71 | -1447.2359 | 0.13334 | 0.71 | -1447.2359 | 0.13333 | 0.71 |
| *ndh*D | -2292.9690 | 0.1726 | -2292.9690 | 0.00009 | 0.99 | -2292.9690 | 0.00009 | 0.99 | -2292.9690 | 0.00009 | 0.99 | -2292.9690 | 0.00009 | 0.99 |
| *psa*C | -349.5986 | 0.0529 | -349.5982 | 0.00073 | 0.98 | -349.5982 | 0.00073 | 0.98 | -349.5982 | 0.00073 | 0.98 | -349.5982 | 0.00073 | 0.98 |
| *ndh*E | -425.4264 | 0.0001 | -425.4264 | 0.00003 | 1.00 | -425.4264 | 0.00002 | 1.00 | -425.4264 | 0.00003 | 1.00 | -425.4264 | 0.00003 | 1.00 |
| *ndh*G | -765.1107 | 0.0967 | -765.1107 | 0.00002 | 1.00 | -765.1107 | 0.00002 | 1.00 | -765.1107 | 0.00002 | 1.00 | -765.1107 | 0.00002 | 1.00 |
| *ndh*I | -700.9534 | 0.0448 | -700.9534 | 0.00006 | 0.99 | -700.9533 | 0.00002 | 1.00 | -700.9534 | 0.00006 | 0.99 | -700.9534 | 0.00006 | 0.99 |
| *ndh*A | -1575.4316 | 0.1472 | -1574.2295 | 2.40413 | 0.12 | -1575.4319 | 0.00062 | 0.98 | -1575.4316 | 0.00004 | 0.99 | -1575.4316 | 0.00005 | 0.99 |
| *ndh*H | -1721.5098 | 0.0966 | -1721.5097 | 0.00012 | 0.99 | -1721.5097 | 0.00012 | 0.99 | -1721.5097 | 0.00011 | 0.99 | -1721.5097 | 0.00011 | 0.99 |
| *rps*15 | -431.49289 | 0.4221 | -431.4929 | 0.00003 | 1.00 | -431.4929 | 0.00003 | 1.00 | -431.4929 | 0.00003 | 1.00 | -430.9005 | 1.18476 | 0.28 |
| *ycf*1 | -9007.5362 | 0.8148 | -9007.5363 | 0.00011 | 0.99 | -9007.5362 | 0.00009 | 0.99 | -9007.5362 | 0.00009 | 0.99 | -9006.0893 | 2.89376 | 0.09 |

#### Table S7. Log-likelihoods and parameter estimates under site models (M1a, M2a, M7 and M8) derived from separate analysis of the protein-coding genes in LSC, IR and SSC regions

| Region | Model | lnL | Estimates of parameters | Positively selected sites | |
| --- | --- | --- | --- | --- | --- |
|  |  |  |  | Amino acid# | *p*-value |
| LSC | M1a  (neutral) | -63880.9263 | p0=0.8478, p1=0.1522  $\omega_{0}$=0.0000, $\omega_{1}$=1.0000 | Not allowed | |
|  | M2a  (selection) | -63877.3139 | p0=0.9942, p1=0.0002,  p2=0.0057  $\omega_{0}$=0.1162, $\omega_{1}$=1.0000,  $\omega_{2}$=9.9564 | 2314 S  3212 V  3772 Q  10893 H  13713 L  14414 P | 0.95*  0.95*  0.99**  0.96*  0.99**  0.97* |
|  | M7  (beta) | -63882.3828 | p=0.0144, q=0.0813 | Not allowed | |
|  | M8  (beta&$\omega$) | -63877.3142 | p=13.0332, q=98.4895  p0=0.9943, p1=0.0057  $\omega_{s}$=9.9939 | 3772 Q  10893 H  13713 L  14414 P | 0.99**  0.95*  0.99**  0.96* |

| IR | M1a | -14561.9960 | p0=0.4249, p1=0.5751  $\omega_{0}$=0.0000, $\omega_{1}$=1.0000 | Not allowed | |
| --- | --- | --- | --- | --- | --- |
|  | M2a | -14542.8657 | p0=0.9046, p1=0.0856,  p2=0.0098  $\omega_{0}$=0.1138, $\omega_{1}$=1.0000,  $\omega_{2}$=13.3938 | 14861 G  16183 N  16585 R  16589 T  16591 L | 0.98*  1.00**  0.98*  1.00**  0.97* |
|  | M7 | -14562.4219 | p=0.0119, q=0.0051 | Not allowed | |
|  | M8 | -14542.8659 | p=99.0000, q=49.2228  p0=0.9966, p1=0.0034  $\omega_{s}$=213.9533 | 14861 G  16183 N  16585 R  16589 T  16591 L | 0.98*  1.00**  0.98*  1.00**  0.97* |

| SSC | M1a | -22790.9124 | p0=0.6384, p1=0.3616  $\omega_{0}$=0.0000, $\omega_{1}$=1.0000 | Not allowed | |
| --- | --- | --- | --- | --- | --- |
|  | M2a | -22770.6364 | p0=0.6360, p1=0.3484,  p2=0.0156  $\omega_{0}$=0.2464, $\omega_{1}$=1.0000,  $\omega_{2}$=13.5942 | 21308 G  21468 T | 0.97*  0.95* |
|  | M7 | -22792.2906 | p=0.0050, q=0.0076 | Not allowed | |
|  | M8 | -22770.7151 | p=0.0228, q=0.0430  p0=0.9821, p1=0.0179  $\omega_{s}$=12.3279 | 21308 G  21468 T | 0.97*  0.96* |

#: The value in front of the amino acid is the amino-acid site position coded by all protein-coding genes concatenated in the order of LSC-IR-SSC regions, the same as those in Table S7.

#### Table S8. Likelihood ratio statistics under random site models.

| Models | | 2△LRT | d.f. | *P*-value |
| --- | --- | --- | --- | --- |
| LSC | M1a vs. M2a | 7.2247 | 2 | 0.0270 |
|  | M7 vs. M8 | 10.1374 | 2 | 0.0063 |
| IR | M1a vs. M2a | 38.2606 | 2 | 4.9182×10^-9^ |
|  | M7 vs. M8 | 39.1121 | 2 | 3.2130×10^-9^ |
| SSC | M1a vs. M2a | 40.5520 | 2 | 1.5641×10^-9^ |
|  | M7 vs. M8 | 43.1510 | 2 | 4.2647×10^-9^ |
| All (LSC, SSC, IR) | M1a vs. M2a | 84.334 | 2 | 0.0000 |
|  | M7 vs. M8 | 93.9007 | 2 | 0.0000 |

#### Table S9. Log-likelihoods and parameter estimates under site models (M1a, M2a, M7 and M8) based on the concatenated sequences of all protein-coding genes.

| Model | lnL | Estimates of parameters | Positively selected sites | | Region |
| --- | --- | --- | --- | --- | --- |
|  |  |  | Amino acid# | *p*-value |  |
| M1a | -101728.6868 | p0=0.76680, p1=0.2332  $\omega_{0}$=0.0000, $\omega_{1}$=1.0000 | Not allowed | | |
| M2a | -101686.5199 | p0=0.9046, p1=0.0856,  p2=0.0098  $\omega_{0}$=0.1138, $\omega_{1}$=1.0000,  $\omega_{2}$=13.3938 | 3772 Q  13713 L | 0.98*  0.96* | LSC |
|  |  |  | 16183 N  16589 T  18391 H | 0.98*  0.95*  0.98* | IR |
|  |  |  | 21225 T  21308 G  21468 T  21505 E  21529 K  21862 S  22440 T | 0.98*  0.99**  0.99**  0.98*  0.98*  0.98*  0.98* | SSC |
| M7 | -101733.4725 | p=0.0050, q=0.01550 | Not allowed | | |
| M8 | -101686.5221 | p=0.3404, q=1.4409  p0=0.9899, p1=0.0101  $\omega_{s}$=13.2408 | 3772 Q  13713 L | 0.98*  0.97* | LSC |
|  |  |  | 16183 N  16589 T  18391 H | 0.99**  0.96*  0.99** | IR |
|  |  |  | 21225 T  21308 G  21468 T  21505 E  21529 K  21771 T  21862 S  22440 T | 0.98*  0.99**  0.99**  0.99**  0.99**  0.96*  0.98*  0.98* | SSC |

#: The value in front of the amino acid is the amino-acid site position coded by all protein-coding genes concatenated in the order of LSC-IR-SSC regions, not the actual amnio-acid site position coded by a specific protein-coding gene.

**FIGURE S1** Phylogeny of the four varieties of *T. ciliata*, together with *T. sinensis* and *M. azedarach* as outgroups: (A). One-ratio model where the same *ω* was set for all branches. (B-E). Two-ratio model where the foreground (red line) and background branches (bule line) were set with different *ω* ratios. Likelihood ratio test was applied to testing the difference between one- and two-ratio models.

**
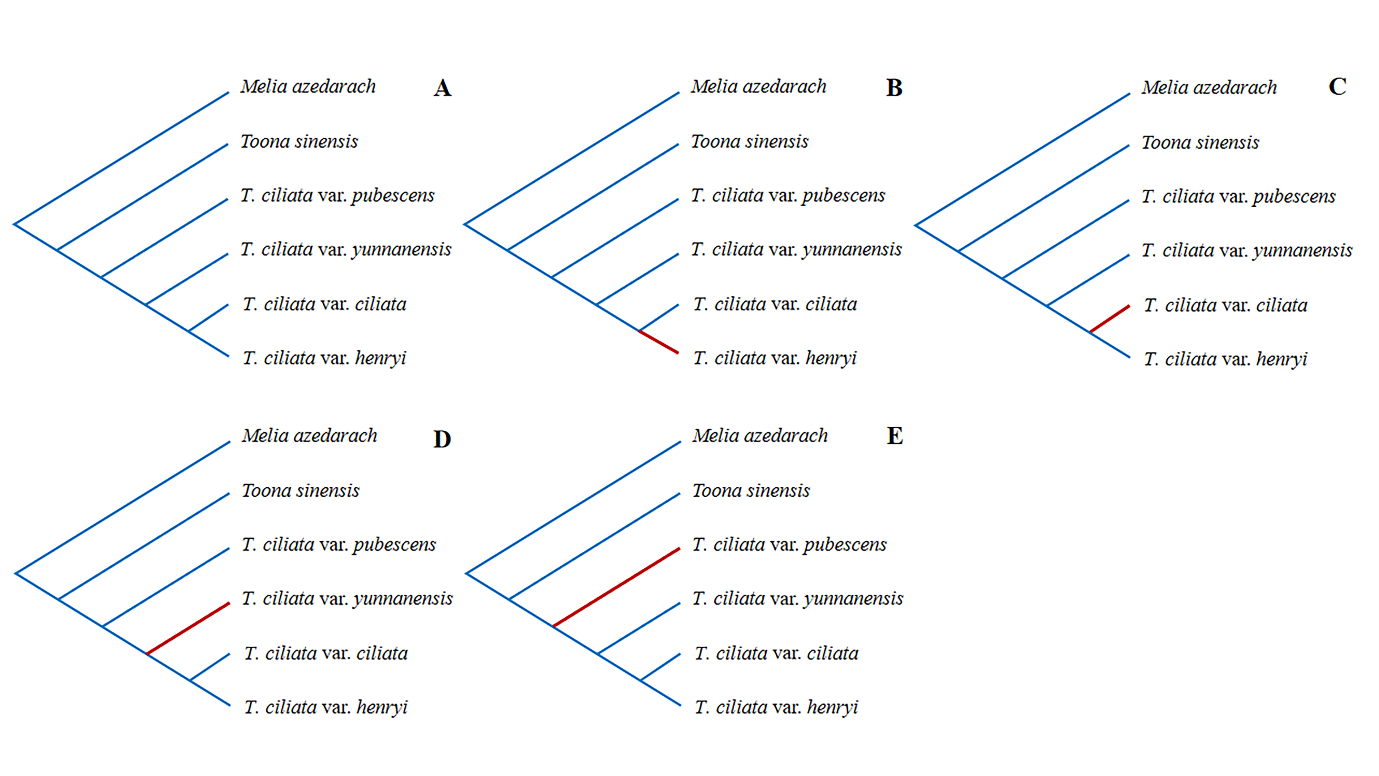
**

**Figure S2**. Comparison of chloroplast genomes among twelve individuals of four varieties of *T. ciliata* using the mVISTA with *T. ciliata* (GenBank access No.: NC_039592) as a reference. The top abscissa line shows the genes in order and the vertical-scale represents the percent identity between 50 and 100% for each individual.


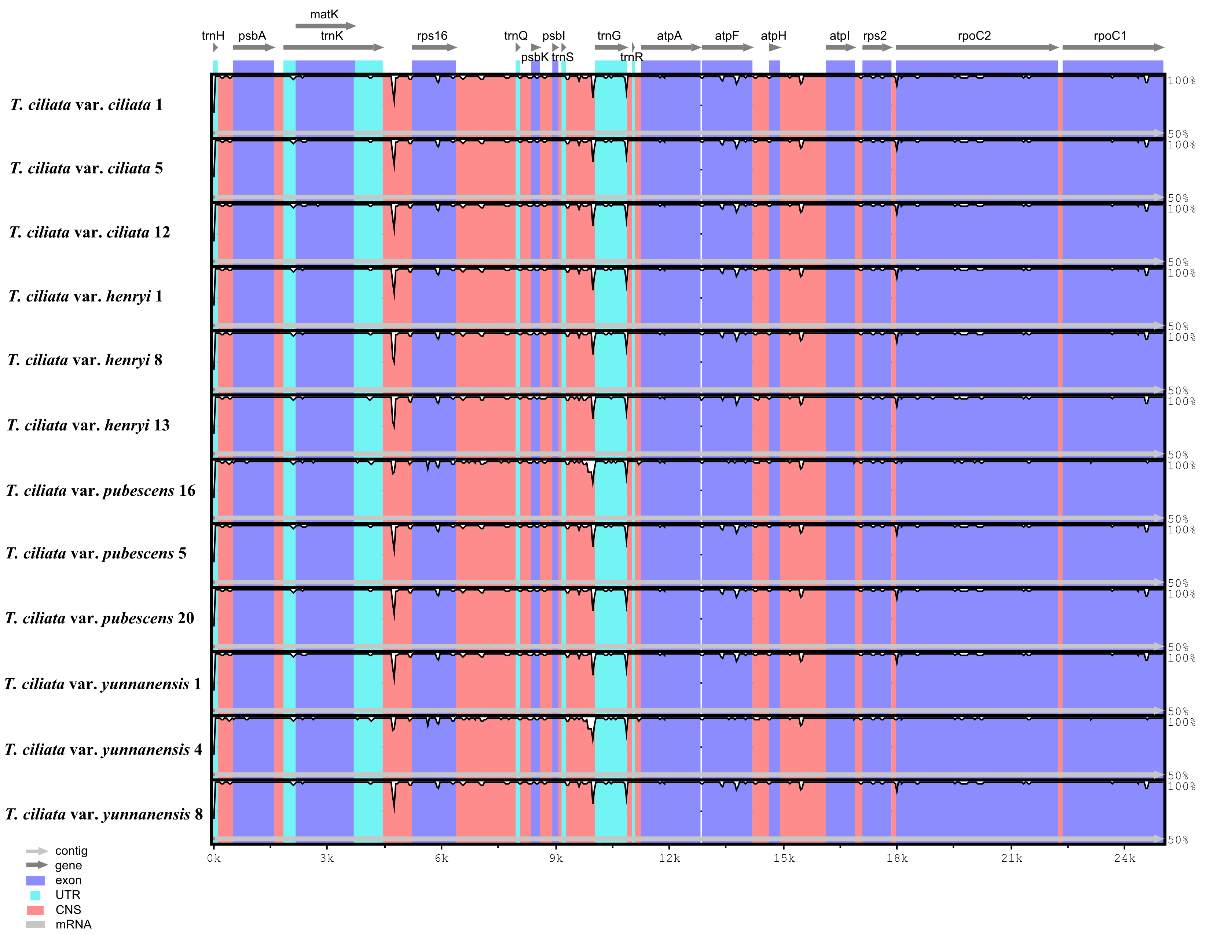


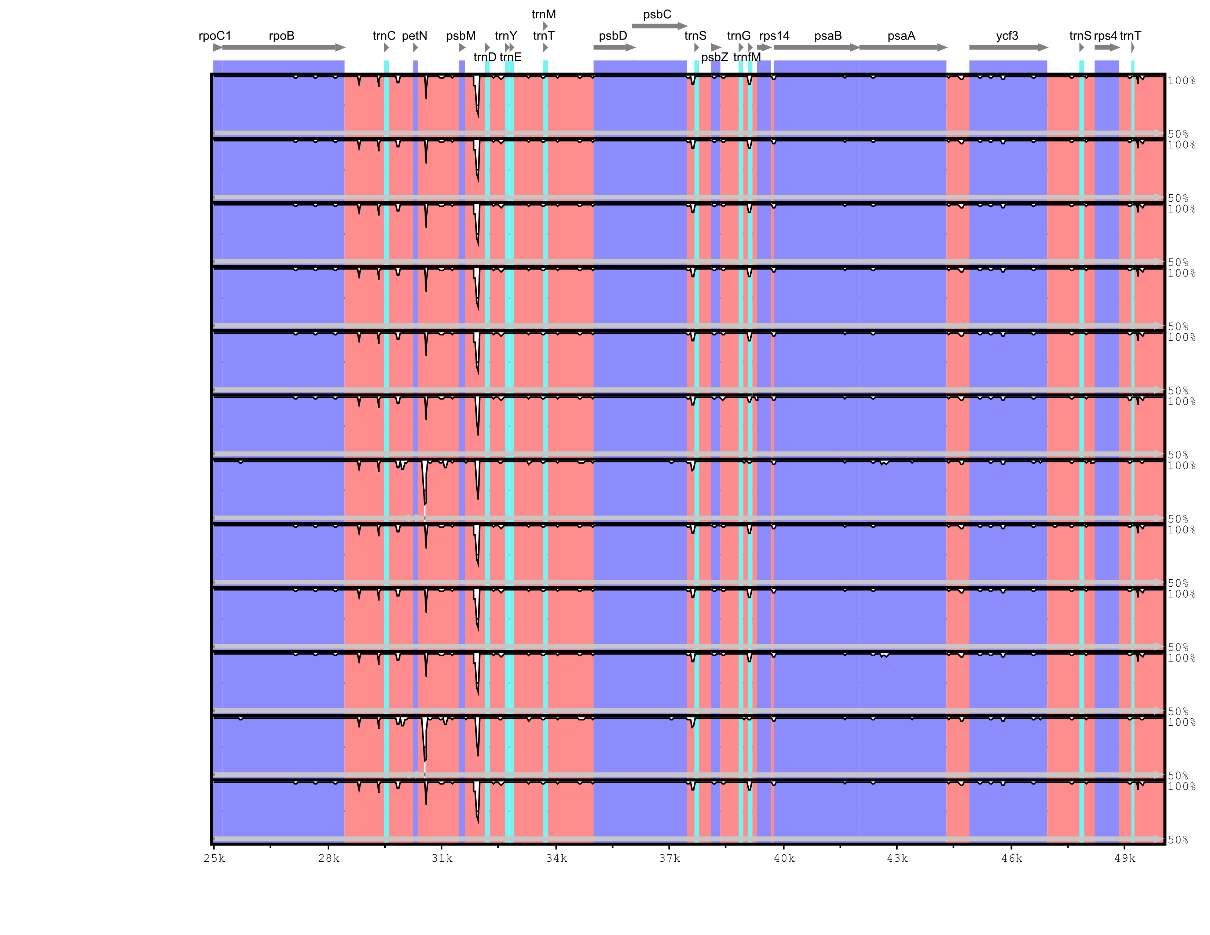


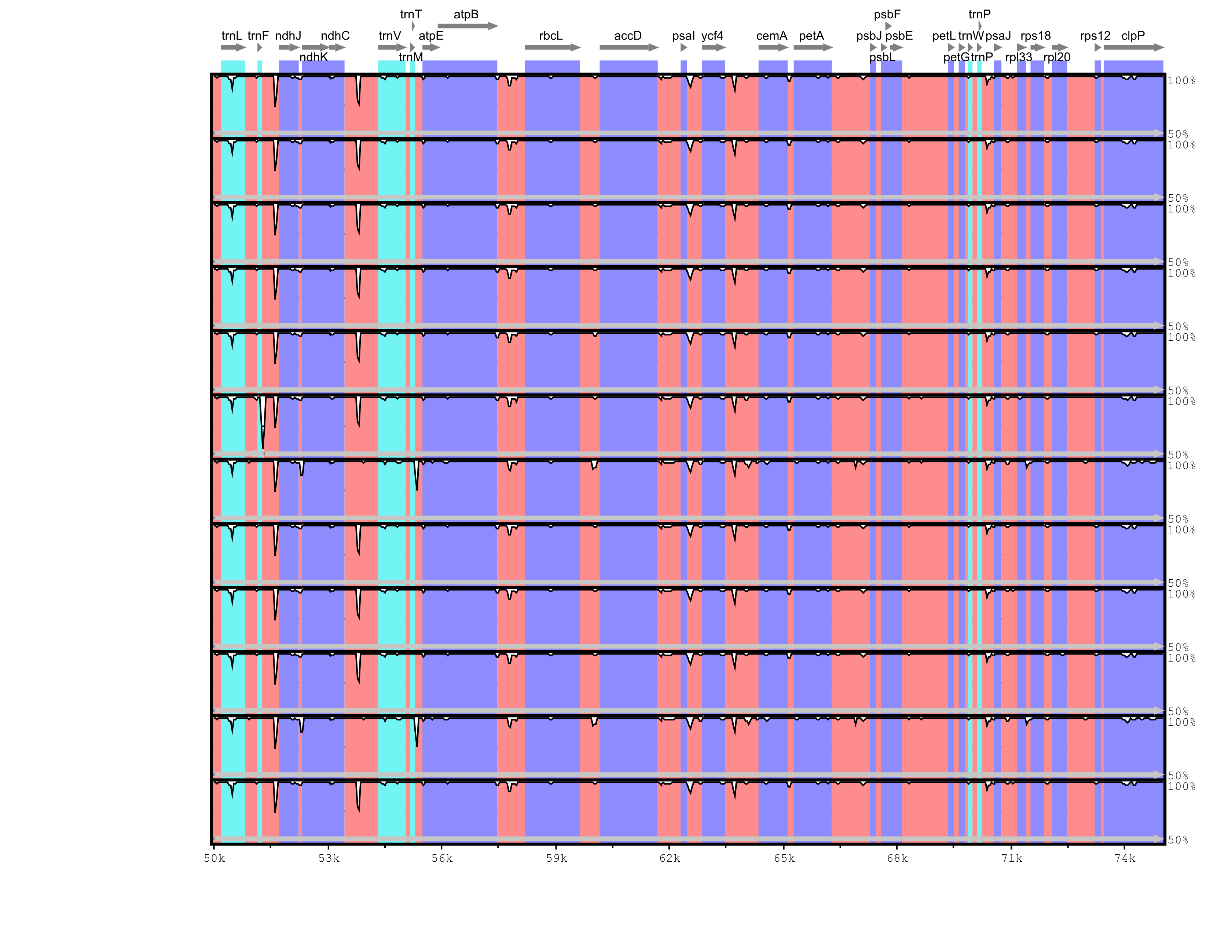


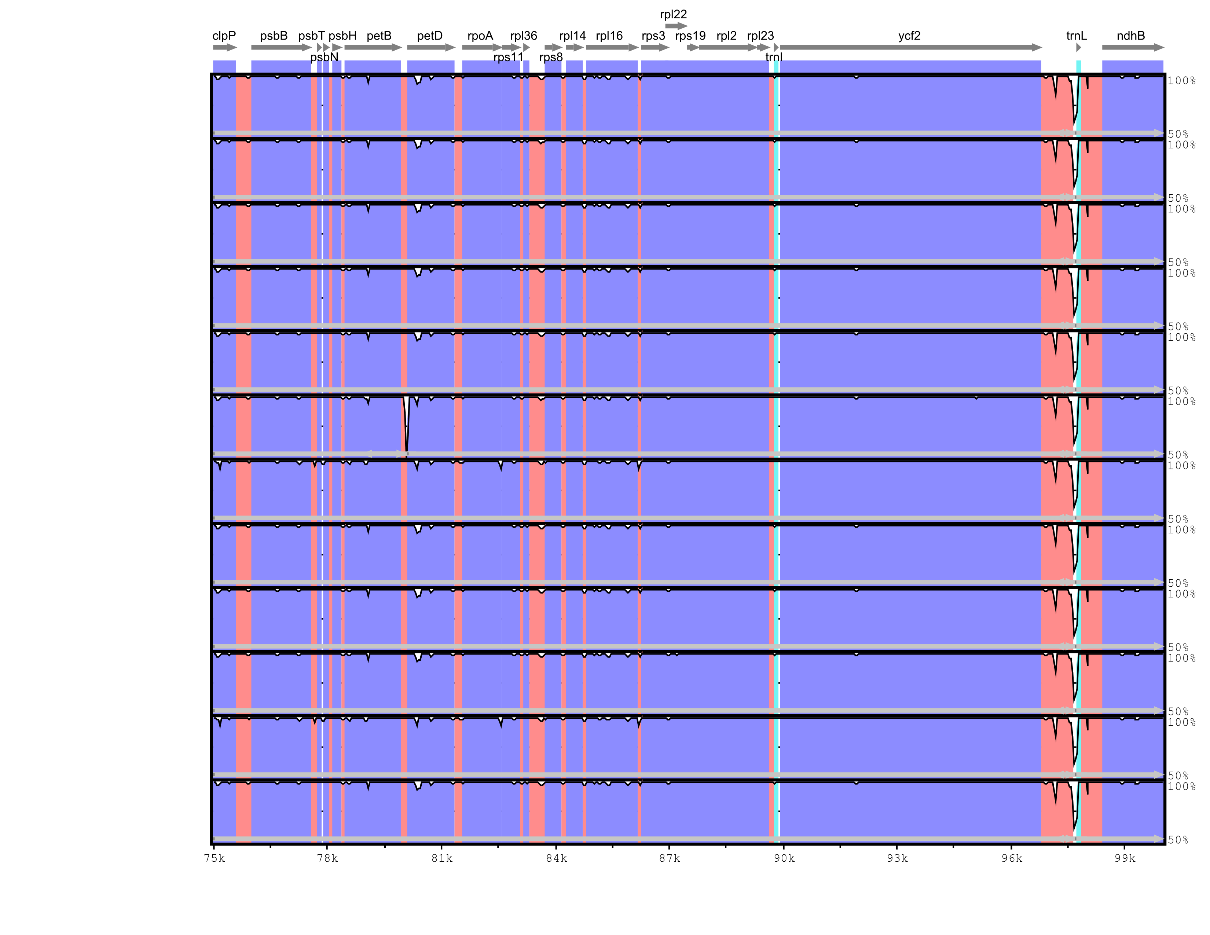


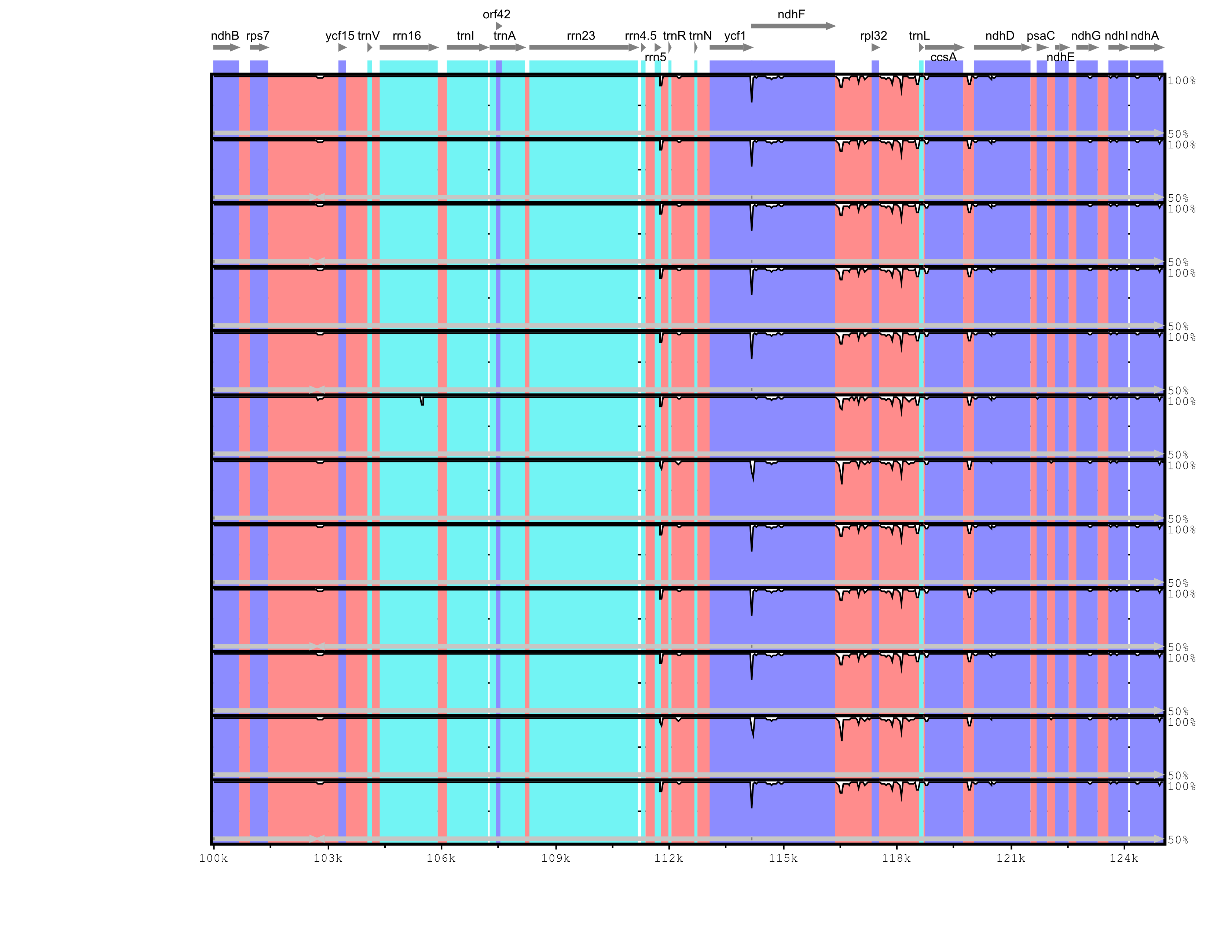


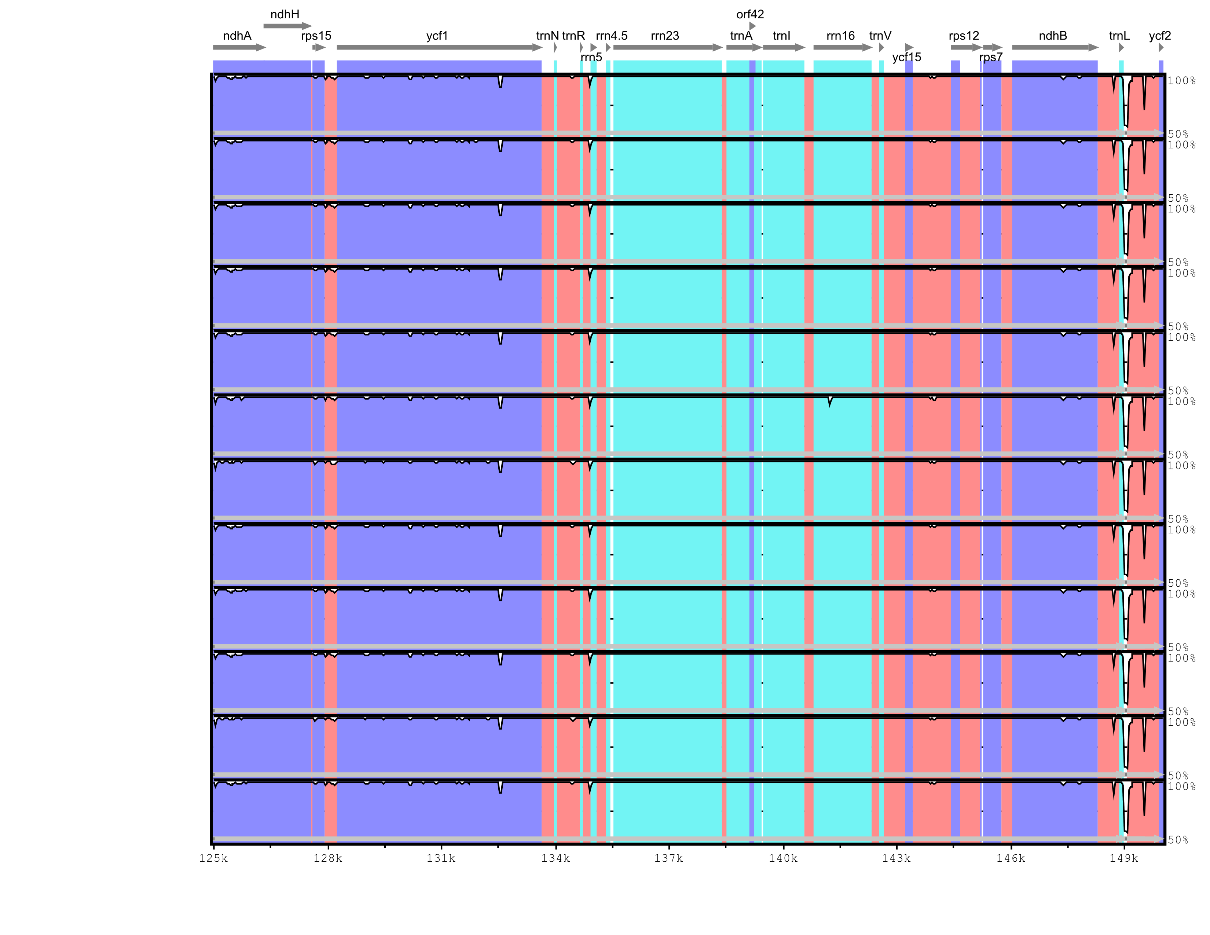


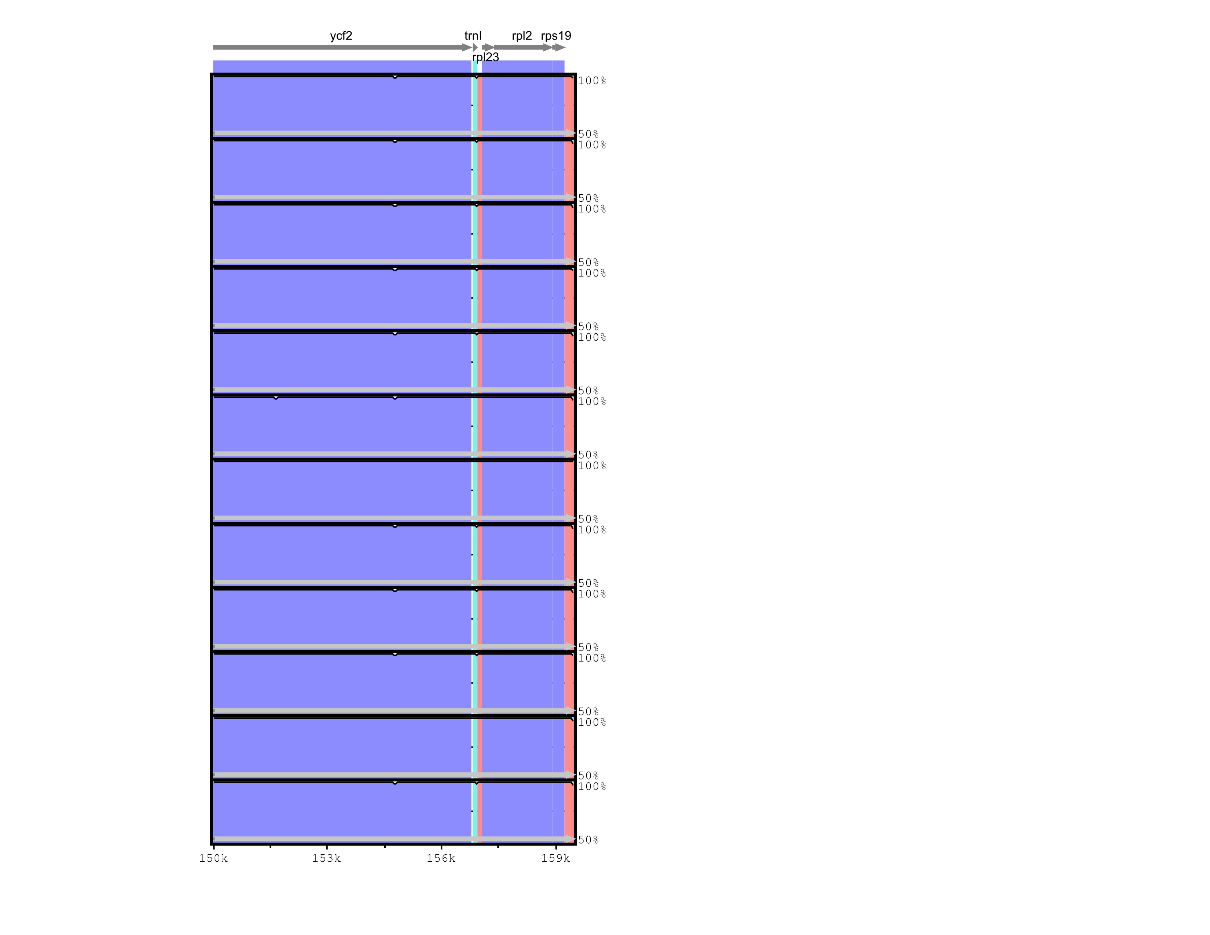


**Figure S3**. Comparison of identified SSR motifs among chloroplast genomes of four varieties of *T. ciliata*.


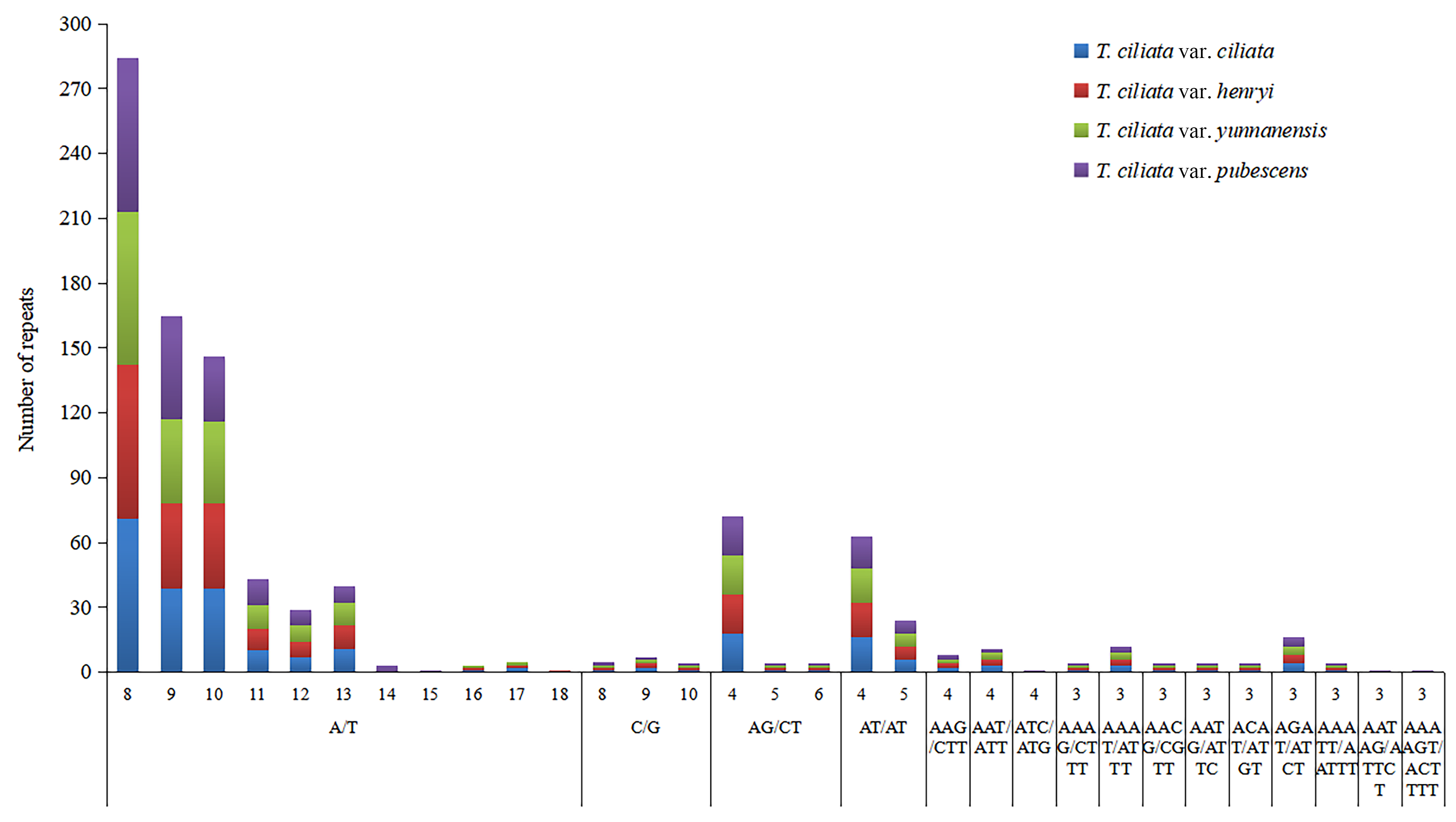


**Figure S4**. Phylogenetic relationship among four varieties derived from the maximum likelihood (ML) method using the concatenated whole genome sequences (only one IR region was included). Branch labels represent bootstrap supporting values of greater than 20%.


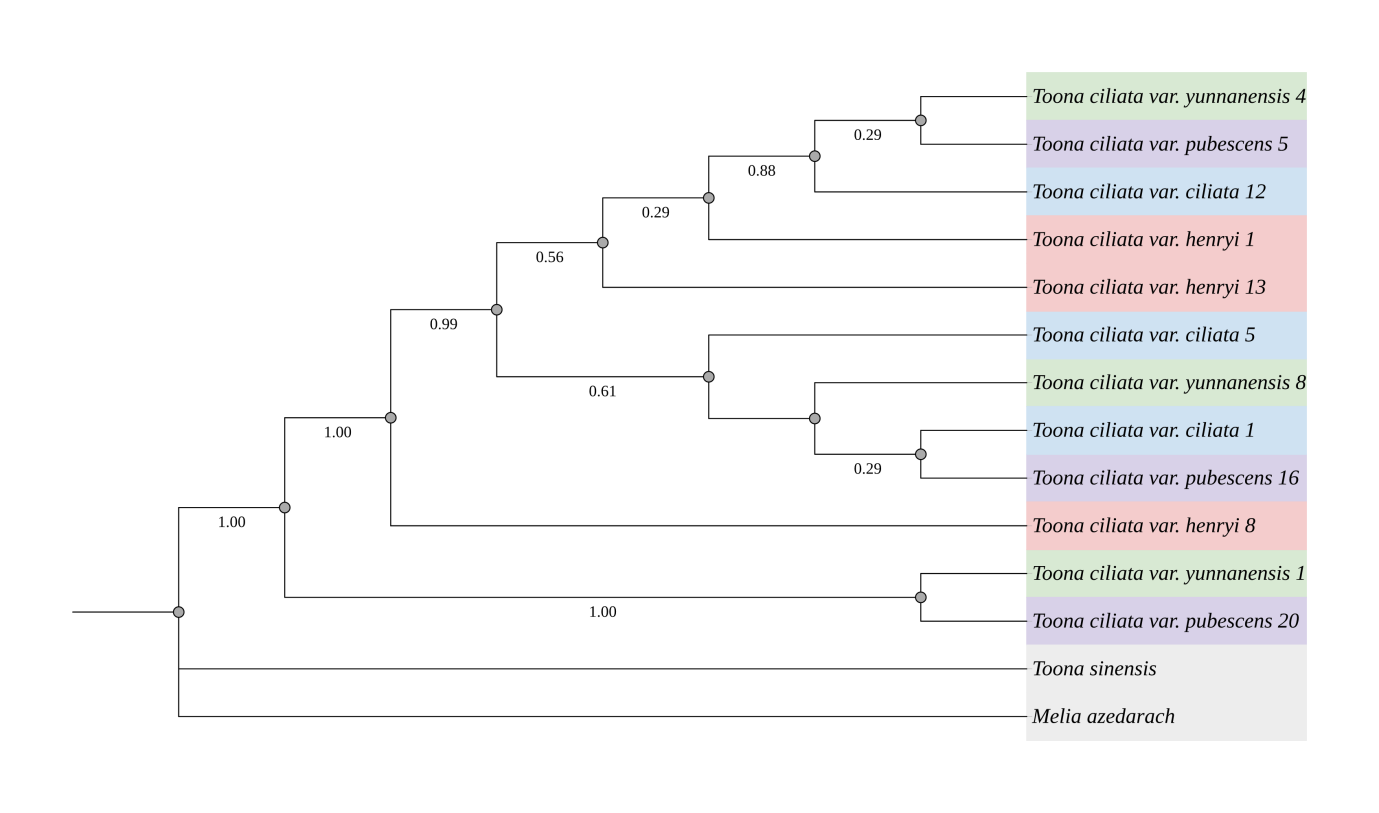


**Figure S5**. Posterior probabilities that each site is from the three site classes ($\omega_{0}$= 0.1138, $\omega_{1}$=1.0000, $\omega_{2}$=13.3938) under the M2a model. The X-axis coordinate is the position of each amino acid site after alignment of 79 protein-coding genes concatenated in the order of LSC-IR-SSC regions.


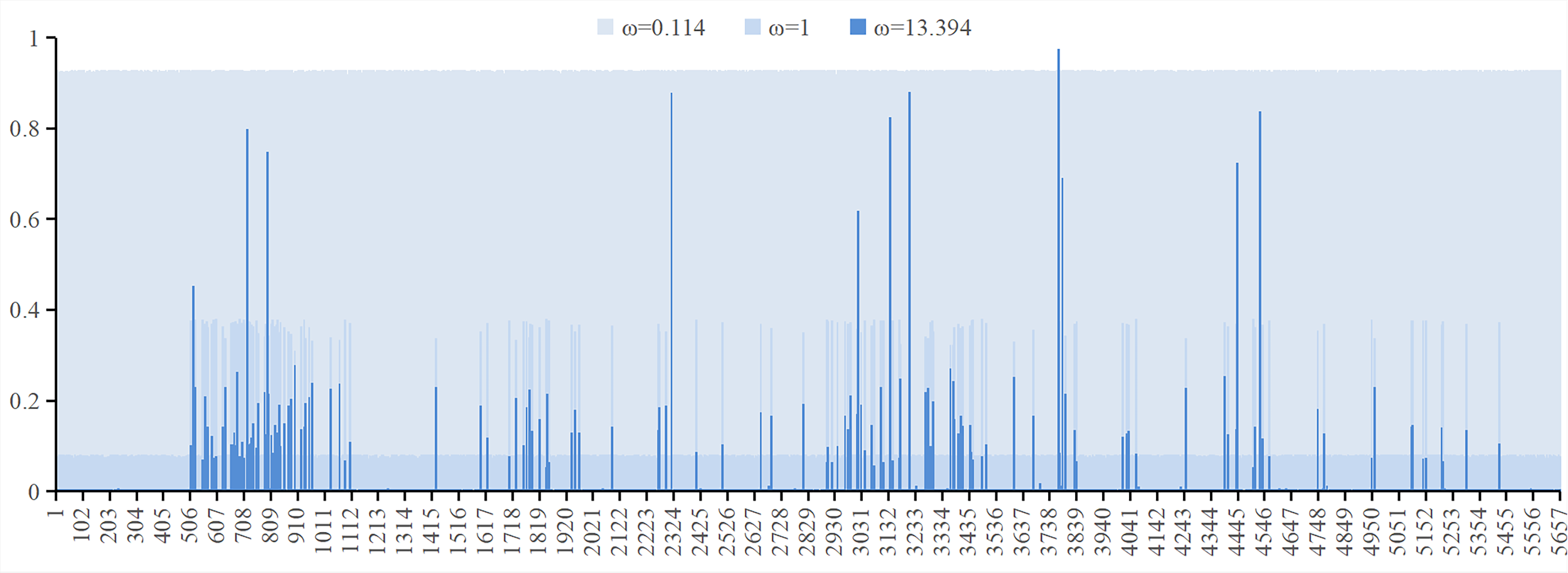

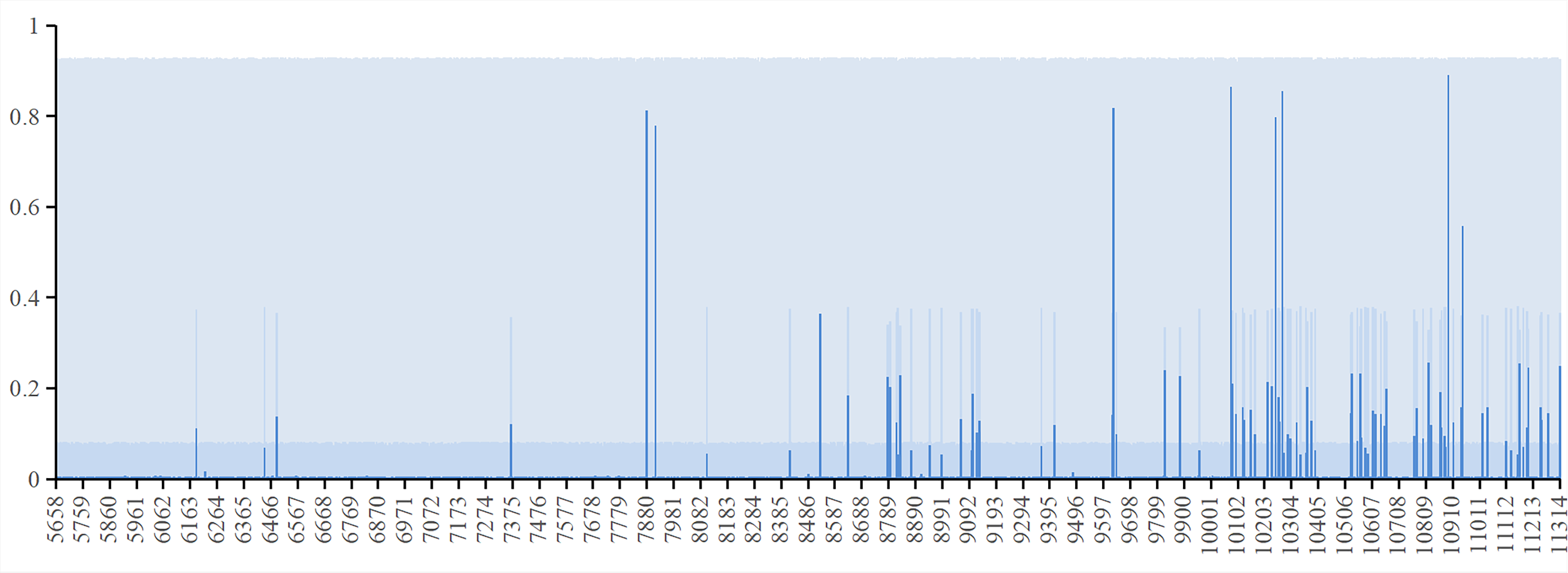

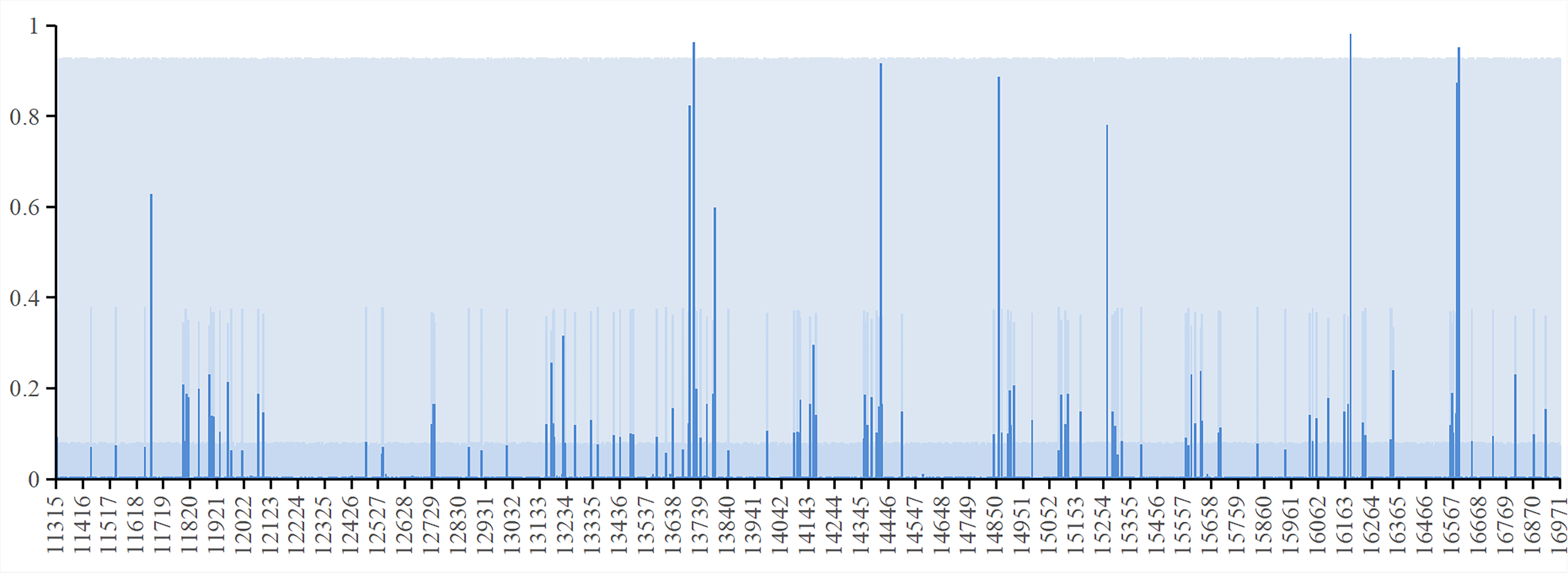

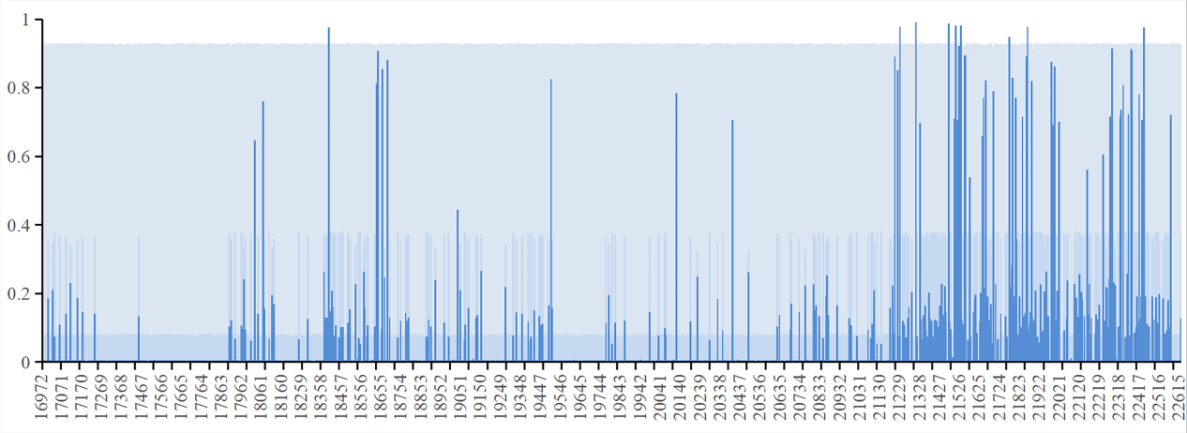

Supplement: Supplementary file 1 — Data S1. [file ECE3-13-e10828-s001.docx]
